# Leveraging family-based assets for Black men who have sex with men in House Ball Communities: Protocol for a cluster randomized controlled trial

**DOI:** 10.1371/journal.pone.0289681

**Published:** 2023-09-08

**Authors:** Jeffrey Birnbaum, Michael Roberson, Marlon M. Bailey, Martez D. R. Smith, DeAnne Turner, Han-Zhu Qian, Sangchoon Jeon, Sabina Hirshfield, LaRon E. Nelson

**Affiliations:** 1 Division of Infectious Diseases, Department of Pediatrics, The Children’s Hospital, State University of New York Downstate Health Sciences University, Brooklyn, New York, United States of America; 2 Center for Race, Religion and Economic Democracy, Union Theological Seminary, New York, New York, United States of America; 3 Department of Women and Gender Studies, School of Social Transformation, Arizona State University, Tempe, Arizona, United States of America; 4 School of Nursing, University of Rochester, Rochester, New York, United States of America; 5 College of Nursing, University of South Florida, Tampa, Florida, United States of America; 6 School of Public Health, Yale University, New Haven, Connecticut, United States of America; 7 School of Nursing, Yale University, New Haven, Connecticut, United States of America; 8 Department of Medicine, STAR Program, State University of New York Downstate Health Sciences University, Brooklyn, New York, United States of America; 9 Yale Institute for Global Health, School of Public Health, New Haven, Connecticut, United States of America; World Health Organization, SWITZERLAND

## Abstract

Black men who have sex with men (MSM) continue to have the highest incidence of new human immunodeficiency virus (HIV) diagnoses in the United States but are least likely to be engaged in care or to be virally suppressed. Many Black MSM face multiple stigmas, but some have found refuge in the House Ball Community (HBC)—a national network of Black lesbian, gay, bisexual, and transgender kinship commitments that provide care-giving, affirmation, and survival skills-building for its members. We propose to modify a skills-building and HIV prevention best-evidence, group-level intervention for HIV- negative Black MSM (Many Men Many Voices) into a family-based intervention to focus on asset-building for both HIV-negative and HIV-positive Black MSM within HBC families. The adapted intervention will be re-branded as Our Family Our Voices (OFOV). We proposed a mixed-methods study to test the feasibility and preliminary efficacy of OFOV adapted for HIV status-neutral use with HBC families. First, we will develop the intervention protocol using the ADAPT-ITT model for modifying behavioral interventions. Then, we will conduct a cluster randomized controlled trial with six HBC families in New York City. Families will be randomized to the OFOV intervention or waitlist control arm. Primary outcomes will be HIV testing, HIV pre-exposure prophylaxis use, currently in HIV care and on HIV treatment. Secondary outcomes will be the number of family-based assets, resilience, number of sexual partners, and relative frequency of condomless anal intercourse. The results of the formative research, including the pilot trial, will contribute to the evidence-base regarding the development of HIV status-neutral interventions that respond to the diversity and complexities of HBC families and that recognize the importance of asset-building for facilitating HBC resilience to stigma as a part of the United States’ domestic policy objective of ending the HIV epidemic by 2030.

## Introduction

HIV continues to disproportionately affect Black communities in the United States (US). Current epidemiologic data indicate that Black individuals represent 42% of new HIV diagnoses, while representing only 13% of the US population [1]. Most new HIV diagnoses among Black individuals have been observed in men who have sex with men (MSM) [2]. Among Black males, male-to-male sexual contact accounted for 79% of new HIV diagnoses in 2019. It is estimated that intragroup transmission accounts for over half of the new HIV diagnoses via condomless anal intercourse (CAI) among Black males [1]; however, the likelihood of HIV risk exposure is influenced by a variety of socioecological factors [3–9].

For many Black MSM, the increased exposure to HIV risk is driven by multiple socioecological factors [9–12]. Some Black MSM seek refuge from environments that disaffirm and/or attempt to dismantle essential aspects of their personhood, including their gender expression(s) and sexualities [13, 14]. Nonetheless, exposure to disaffirming environments has been reported to have residual negative psychosocial impacts [15–17]. In our previous work, we found that early-life (before age 18) exposure to environments that disaffirmed same-gender sexual behaviors was associated with an increased number of sex partners and HIV diagnosis in adulthood [18]. In a longitudinal study [19], we also found that financial crisis was associated with reporting a greater number of male sexual partners in the past 6 months; a recent conviction was associated with a sexually transmitted infection (STI) diagnosis at 6-month follow-up; and unstable housing was associated with an STI diagnosis at 12-month follow-up. Thus, the absence of “behavioral risk factors” does not confer protection against other economic and social hardships that still exert influence on HIV outcomes for Black MSM. It is therefore necessary to identify and strengthen assets to support resilience to hardships that can undermine the effects of HIV interventions.

The House Ball Community (HBC)—a national network of Black lesbian, gay, bisexual, and transgender persons (LGBT) kinship commitments (families)—provides caring, affirmation, and survival skills-building for its members [14]. The HBC is a thriving community within the larger Black and Latinx LGBT communities. The HBC is organized to meet the needs of its members for social solidarity and mentoring in a society largely hostile to diversity in sexual and gender expression [20]. The term “houses” refers to the actual familial networks, which are generally not localized in any one place or residence. The “ballroom” or “balls” are the common terms for competitive dance and performance events that occur at regular intervals throughout the year and constitute an event circuit [21]. Each House structure has a “father” and a “mother” who assume the traditional roles performed by parents of origin regardless of gender, sex or age, and act as surrogate parents to the House. Generally, parents provide a safe haven and source of support for their house members in situations where they have been rejected by their families of origin or other social institutions [20]. The HBC population size is estimated to be approximately 8,000 nationwide, with the largest concentration in New York City [21]. Despite the emerging visibility and celebration of the HBC’s contributions to culture in the Black LGBT communities, alarming disparities in HIV persist. Previous studies with HBCs identified HIV prevalence as high as 20% [22], as well as a high prevalence of stigma and history of stressful life events [22, 23]. Moreover, Black HBC members were 2.5 times more likely than non-Black Latinx members to be unaware of their HIV status [23].

Many Men Many Voices (3MV) is a multi-session, group-level behavioral intervention developed to prevent HIV and STIs among Black MSM. A summary of the 3MV sessions is presented in Table 1. The intervention is designated as a best-evidence intervention by the U.S. Centers for Disease Control and Prevention for its demonstrated efficacy in improving rates of HIV testing and reducing the frequency of CAI [24, 25]. Although 3MV has strong evidence for reducing HIV/STI behavioral risk in the general population of Black MSM, it was not designed to specifically respond to the social and cultural dynamics of HBC family life that are realities for some Black MSM. To optimize the HIV prevention outcomes for Black MSM in HBC families, we sought to go beyond identifying and reducing risk factors towards simultaneously identifying, increasing, and supporting assets that are present in HBC family life.

**Table 1:** Summary of original 3MV intervention modalities, session topics and lengths.

Modalities	Sessions	Length (Mins)
▪ Small group activity ▪Brainstorms ▪ Role plays ▪ Paired work ▪ Develop menu of prevention options ▪ Action planning	1. MSM and dual identity	140
	2. HIV/STD prevention for MSM: the roles and risks for tops and bottoms	210
	3. HIV/STD risk assessment and prevention options	110
	4. Intentions to act and capacity for change	170
	5. Relationship issues: partner selection, communication, and negotiation of roles	140
	6. Social support and problem-solving to maintain change	125
	7. Building bridges and community	130

Internal and external assets are key influencing factors in the health-seeking behaviors of MSM. Building internal social assets as part of family-based socioecological assets is critical to facilitating engagement in health-promoting behaviors [26–32]. External assets are defined as relationships, support systems (e.g., family support), and opportunities such as positive peer influence and creative activities [33–35]. Internal assets are skills and personal values such as self-esteem, a sense of purpose, positive identity, and planning/decision-making capacities [35, 36]. Although it is important to have interventions that focus on reducing behavioral risk for HIV, it is important that Black MSM be able to build interpersonal-level (peer-group/family) and intrapersonal-level (individual) capacities to challenge stigma, recover from stigmatizing experiences, and use strategies to protect against the internalization of stigma that may result from chronic exposures to stigma and social hardships [37, 38]. While limited data exist on assets among Black LGBT youth, available evidence indicates that LGBT youth generally have fewer assets than heterosexual youth. For example, in a population-based survey of assets among youth in grades 6–12, compared to non-LGBT youth, smaller proportions of LGBT youth had assets related to family support, positive family communication, safety, feeling valued in the community, family boundaries, adult role models, and youth programs [39]. The only asset with a higher prevalence among LGBT youth was engagement in creative activities. This intersects with the substantial existing evidence that LGBT youth of color have worse outcomes compared to the general population of LGBT youth [40–42]. To date, there are no known group-level, HIV behavioral risk-reduction interventions that also incorporate building family-based socioecological assets to optimize prevention and treatment outcomes for Black MSM. For this reason, 3MV is being adapted to be more responsive to the sociocultural realities of HBC life and to include family-based assets. The primary aims of this study are to: (1) conduct formative research to identify key modifications to the 3MV intervention manual and implementation protocol and (2) conduct a pilot cluster randomized controlled trial (CRCT) to determine the feasibility and acceptability of the modified intervention manual and trial protocol.

## Materials and methods

### Study design and setting

**Fig 1:**
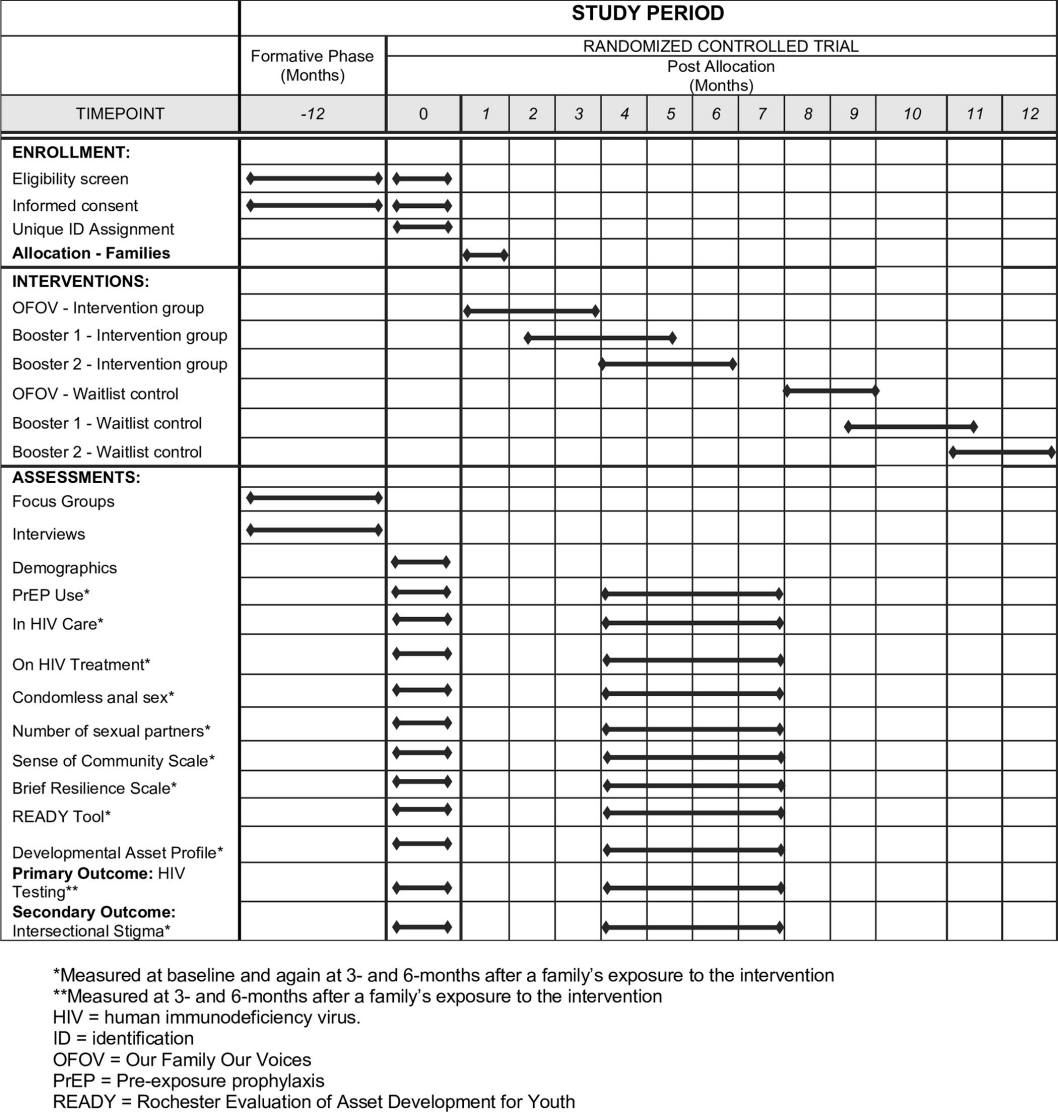
Schedule of enrollment, interventions, and assessments.

**Fig 2:**
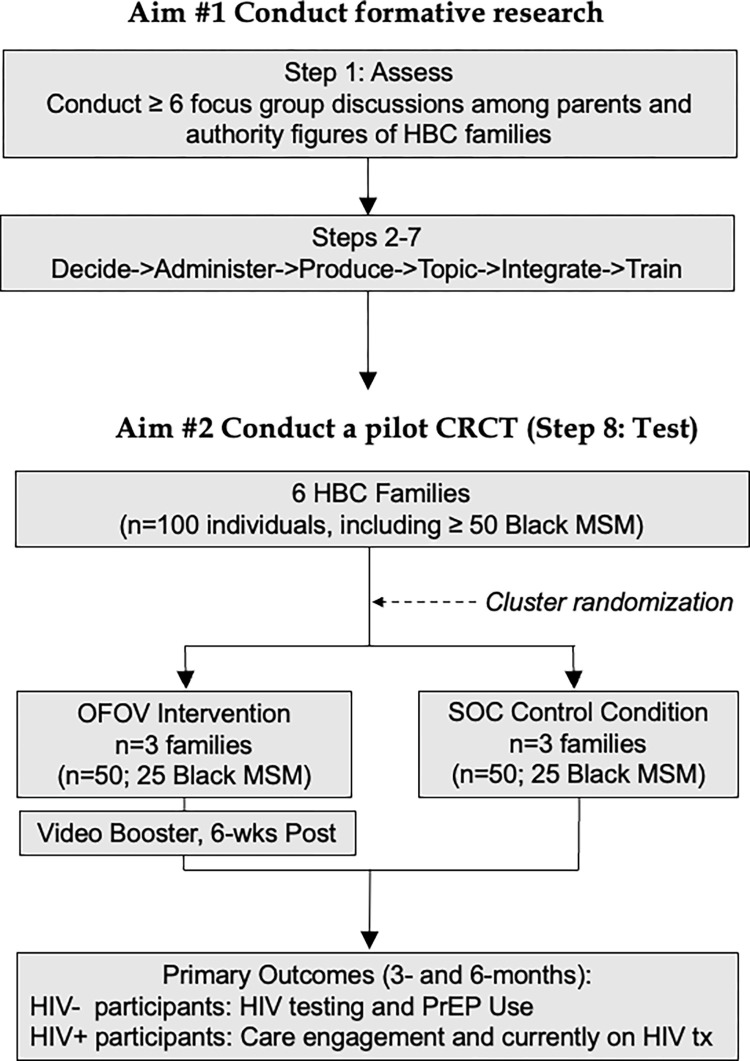
Study design.

The description of the study protocol conforms to the specifications of the Standard Protocol Items Recommendations for Intervention Trials (SPIRIT) checklist (include as a S1 File). This study consists of a formative phase and a trial phase, as detailed in Fig 1 and depicted in Fig 2. We will use a mixed-methods approach to investigate our study aims. First, we will use qualitative (focus group discussions [FGDs] and interviews) methods to determine what HBC family-specific adaptations need to be made to the original intervention. We will then conduct a CRCT (quantitative) to determine the feasibility and acceptability of the trial protocol as well as to estimate the preliminary effect size of the OFOV intervention on HIV testing, PrEP use, engagement in HIV care and current use of antiretroviral therapy for HIV treatment. The ADAPT-ITT model is a systematic framework that outlines a step-by-step process for researchers and implementers to follow when translating existing HIV behavioral interventions for use with different populations [43, 44]. A modified ADAPT-ITT model will guide our approach to enhancing 3MV’s content to incorporate an asset-building framework that leverages the strengths of the families. The framework has been used in previous studies, including a study adapting 3MV for use with MSM in Ghana, West Africa [45]. Aim 1 will encompass ADPAT-ITT steps 1–7, leading to the combined multi-level intervention adapted to improve congruence with the realities of HBC family life. Although our previous programmatic experience already provided us with evidence to know what our selected intervention of focus will be, and that it requires adaptation for HBC (usually ADAPT-ITT Step 2), we will still leverage the ADAPT-ITT model to systematically guide our adaptation process. Aim 2 encompasses step 8, in which we will assess the feasibility and acceptability of conducting a CRCT of *OFOV* with a standard of care (SOC) wait-listed control condition. The study will be conducted in New York City (NYC), which is the geographic area with the highest concentration of individuals in the HBC [21].

### Sample size

For Aim 1, we will conduct six FGDs with parents of HBC families. We anticipate that 6–8 parents or parental authority figures will participate in each FGD. Thus, the estimated sample size is between 24–48 parents. Based on our many years of experience working within the HBC and our geographic concentration in the NYC metropolitan area (inclusive of northern New Jersey and southern Connecticut), we do not expect a wide variation in responses between the focus groups; therefore, we expect that we will reach data saturation by the fourth focus group.

For Aim 2 we will recruit six HBC families (n = 100). By assuming an intraclass correlation coefficient (ICC) of 0.01 within a family, a design effect of 1.12 was adjusted for the power analysis. This sample size will provide 80% power to detect a medium effect size of 0.6 on stigma at a 5% significance level. In a previous study performed for 3MV, they found a significant odds ratio of 1.81 on HIV testing at 6-month follow-up after adjusting for approximately 40% sample reporting engaging in HIV testing in the past 3 months before the intervention. In the proposed trial, the sample size of 100 will allow us to detect a large odds ratio of 3.30 with 80% power and could estimate a proportion of HIV testing with 14% standard error.

### Participant eligibility

For Aim 1, individuals are eligible to participate in the FGDs if they are at least 18 years old, are the publicly recognized parents or authority-figures of an HBC family, and their immediate house family-unit has at least twelve members. Individuals will be excluded if their HBC family has been established for less than 12 months and the person lives outside of the NYC metropolitan area. For Aim 2, eligibility for the study will be determined primarily at the family-level. At least 12 members of the same HBC family must be willing to participate together, of which at least half the participating members must be Black MSM. Participating family members must reside in the NYC metropolitan area. Black MSM participants must be at least 18 years old, currently self-identify as cisgender or transgender man, and report a history of anal sex with another man at least once within the previous six months.

### Recruitment

To achieve Aim 1, the research study coordinator will work with our HBC partners to conduct a chain-referral method to recruit the sample. This process involves the research study coordinator working with the HBC partners to identify and recruit house parents. HBC partner parents will be asked to invite other parents from their social network to participate in FGDs. HBC partner parents will also be asked to invite members of their own families whom they deem authority figures to other house members. In addition to the chain referral method, a flyer advertising FGDs will be circulated on various social media platforms instructing HBC members to contact the research coordinator via email for additional information about the study. The research coordinator will then screen parents for eligibility via a telephone and then store the screening information in a locked spreadsheet on the State University of New York (SUNY) Downstate Health Sciences University server. To minimize potential confidentiality and privacy breaches, all recruitment screening calls will be conducted at in a SUNY Downstate Health Sciences University clinic.

To achieve Aim 2, we will recruit six HBC families (n = 100). We will conduct recruitment using two methods. First, we will disseminate recruitment/marketing materials via HBC online communication platforms. Potential participating families can contact the study office directly via text message or phone call in response to marketing materials. Second, we will recruit through word-of-mouth referrals from our HBC community partners and other outreach activities.

### Study measures

#### Biomarker measures

We will only collect biomarker measures of participants in the CRCT phase of the study (Aim 2). Participants who are HIV-negative will have the option of taking a home HIV testing kit to screen for HIV antibodies at the baseline appointment. Participants will also have the option of getting a home HIV testing kit or DBS at the 3- and 6-month follow-up points. Participants will be expected to take a photo of their home HIV test kit results and upload the photo to a password protected server within 2 weeks of taking their surveys. Participants who are HIV-positive will have the option of getting their viral load assessed via dry blood spot (DBS). Houses that are randomized to the SOC waitlist control arm will not be exposed to the OFOV intervention during the 6-month data collection period; however, they will be offered the same opt-in testing for HIV or DBS for viral load. For individuals whose HIV test reveals an undiagnosed HIV infection, the study staff will offer to link the individual to follow-up HIV primary care at SUNY Downstate Health Sciences Center for rapid start of HIV antiretroviral (ARV) treatment. For individuals who were previously diagnosed with HIV, the study coordinator will ascertain whether the individual is currently retained in care and, if not, offer to help re-connect the individual to HIV primary medical care services.

#### Self-report measures

The self-report measures for the CRCT phase of the study (Aim 2) are summarized in Table 2. Our primary outcomes will be HIV testing and PrEP use (for HIV-negative or HIV status unknown members) or “In HIV care” and “currently on HIV treatment” for people living with HIV (PLHIV) within 6 months of randomization. For HIV-negative MSM we will also record the frequency of HIV testing over the prior 3-months; however, we anticipate that few participants will take >1 test in this short period of time. Our secondary outcomes will be the number of family-based assets, resilience, number of sexual partners, and relative frequency of CAI. We will also assess sense of community [46] at all time points. In our previous research with Black MSM, we found that sense of community was associated with increased condom use for anal intercourse (*OR* = 1.26, 95%CI 1.05,1.52; p < .05) [47]. We will characterize the sample on intersectional stigma by creating a latent variable that is drawn from three scales that measures HIV stigma, same-sex stigma, and gender non-conforming stigma. For HIV negative MSM, the latent intersectional stigma variable will exclude the enacted and internalized subscales of the HIV stigma scale because those are designed for PLHIV. We will also assess implementation outcomes of feasibility and acceptability.

**Table 2:** Summary of aim 2 CRCT phase measures.

CONSTRUCT	SAMPLE ITEMS
**HIV testing** (self-report and opt-in testing results)	Number of HIV tests in past 3-months
**PrEP use** (self-report)	Is participant currently on antiretrovirals for HIV PrEP?
**In HIV care** (self-report)	Has participant attended HIV medical appointment in past 3-months
**On HIV treatment** (self-report and opt-in DBS testing)	Is participant currently on antiretrovirals for HIV treatment?
**Condomless anal intercourse** (past 3 months)	Relative frequency = # times condoms used during anal sex / total # episodes of anal sex
**Number of sexual partners**	Total number of sexual partners in the past 3 months
**Sense of Community Scale [46]** (8-items)	I feel like I am a member of my house family
**Brief Resilience Scale [48]** (6-items)	I tend to bounce back quickly after hard times?
**READY Tool** (24-items) [49, 50]	My family expects me to try my best.
**Developmental Asset Profile (40-items)** [39]	I seek advice and counsel from my parents. My family has clear rules and consequences.
***INTERSECTIONAL STIGMA***	
**HIV Stigma Scale [51]**	
*Enacted subscale (10-items)*	Has a healthcare worker not wanted to touch you because you have HIV?
*Perceived subscale (10-items)*	In your community, how many people think a person with HIV is disgusting?
*Internalized subscale (10-items)*	How often do you feel guilty about having HIV?
**Same-Sex Stigma Scale** [52](10-items)	How often have you lost a place to live for being homosexual?
**Gender Non-Conformity Stigma Scale [53]** (10-items)	How often have you been called names because of your feminine mannerisms?
***IMPLEMENTATION OUTCOMES***	
**Feasibility:** recruitment yield	% of eligible HBC families who decide to participate
**Feasibility:** coordination time	Length of time it takes to coordinate a retreat
**Acceptability:** completion rate	Proportion of participants who complete Our Family Our Voices
**Acceptability**	Overall, I found the sessions helpful
**Acceptability**	The sessions were relevant to me

### Study procedures

#### Step 1 (assess)

We will conduct FGDs with parents and authority figures of HBC families to inform the adaptation of 3MV by deepening our understanding of the range of assets in the external (i.e., support, empowerment, boundaries/expectations, and constructive use of time) and internal domains (i.e., positive identity, positive values, social competencies, and commitment to learning). The FGDs will also explore their observations of the various stigmas and hardships faced by family members, how these stigmas intersect, and their views on what are the best combinations of assets to buffer against the negative effects of intersectional stigma on HIV testing, PrEP use, linkage to care and ARV use. Using the data generated from the FGDs, we will qualitatively characterize the external and internal assets that exist within families, including patterns of commonalities and differences between families, identify sources of exposures to intersectional stigma, and explore the various ways that intersectional stigma influences health goals in HBC families.

#### Step 2 (decide)

As previously noted in the description of our study design, Step 2 of ADAPT-ITT (as originally defined) has already been completed. Our selection of the 3MV intervention for use with HBC families was pre-determined; thus, we will conduct a modified version of Step 2 that will help us to decide which activities to modify. Guided by the formative research results in Step 1, the team will meet to discuss what specific intervention activities should be modified to incorporate family-based assets. We will conduct an updated scoping review of research literature to identify innovative evidence-based options that can be used to enhance focus on assets that will facilitate resilience. We will pay special attention to identifying technology and social media options. This step will also include a review of the current intervention activities and discussion of how they can be refined to identify and build assets against intersectional stigma and hardships. In consultation with HBC partners and a community advisory board, we will incorporate changes into the curriculum, identify gaps/errors in logic and make corrections in preparation to conduct a workshop of the intervention with a reference group of HBC families.

#### Step 3 (administer)

We will use a modified version of Step 3. The original ADAPT-ITT step calls for the original version of the intervention (3MV) to be administered and then, through that administration, identifying what needs to be changed before subsequently producing a first draft of the adaptation in the next Step 4. We will develop a preliminary adapted version of the original intervention manual and administer this adapted version (OFOV) in a series of facilitated simulation exercises with role-play over a 3-day workshop period. Participants will work through each of the intervention components, focusing on the family asset-building content and reviewing our approach to “connecting the dots” that these assets that HBC family members possess are socioecological resources that can buffer against the impact of intersectional stigma.

#### Step 4 (produce)

Based on the feedback from the simulation workshop participants and our observations of their engagement with the activities, we will determine which content and approaches should be incorporated into the *OFOV* intervention manual. This process includes making final decisions on the storyboard and reflective activities. We will retain intervention components most consistent with current scientific evidence on HIV risk-reduction and asset-building, as well as HBC cultural relevance based on input from our HBC partners.

#### Step 5 (topical experts)

We will engage topical experts to review the adapted OFOV manual and provide feedback on its congruence with the original intervention and its immediate local sociocultural relevance. A copy of the adapted intervention manual will also be provided to a co-investigator who is one of the world’s foremost authorities on HBC culture and factors that influence HIV prevention and care in their communities. He will review the manual to ensure that it resonates with ballroom sociocultural norms (e.g., preventing the development of a contrived product that does not elicit a natural, authentic connection with members of the HBC). Paid community consultants will also review the OFOV intervention manual for cultural resonance as well as to ensure that it maintains fidelity to the internal logic of the original 3MV intervention on which it is based.

#### Steps 6 (integrate) and 7 (train)

In these steps, we will receive feedback from the topical experts and summarize it in a report that will be distributed to the entire investigator team, including our key collaborators. We will discuss the feedback and provide follow-up clarifying questions to the topic experts, as necessary, before making final revisions to the adapted intervention manual. We will also produce a training manual to standardize training and permit future replication of the intervention in other settings. We will provide comprehensive training to facilitators whose roles are dedicated to delivering the intervention. We will also develop the training to be multi-faceted to facilitate comprehension and retention of training concepts, using strategies that the PIs have successfully deployed in other HIV prevention research projects [54].

#### Step 8 (test)

Using a CRCT, we will evaluate the feasibility, acceptability, and preliminary efficacy of *OFOV* compared to an SOC waitlist control arm on uptake of HIV testing and PrEP use (for HIV-negative individuals) and linkage to care and current ARV uptake (for PLHIV) among 6 NYC HBC families over a 6-month period. SOC is defined as the currently available suite of prevention and care services in the NYC metropolitan area. Since the HBC is relatively small and recruitment is limited to the NYC metropolitan area, we propose a CRCT design to reduce the likelihood of contamination across families in the HBC. Once a cluster is randomized to the treatment condition, it will remain in the assigned condition for the duration of the study. Using this study design, each cluster (family) in the experimental arm will receive both the OFOV intervention over a 2.5-day weekend retreat and participate in a booster activity session at 6 weeks and 18 weeks post-baseline. The SOC control will not receive the OFOV intervention during the 6-month data collection period. The SOC control, however, will have the option to participate in OFOV after the 6-month data collection period concludes.

#### Booster sessions

Two booster session modules will be delivered at week 6 and week 18 post-*OFOV* intervention. The boosters are group “homework assignments” designed to leverage the families’ creative talents to conceptualize a future life story that centers on the strengths of the family, while simultaneously utilizing information, principles, and skills from the OFOV retreat. The first booster will involve the drafting of story boards, which allow each participant to draft a new “story” that reflects their individual vision for their lives. The second booster builds on the storyboard booster by having the family self-produce a short [5-minunte] video (using basic smartphone technical capabilities) that portrays a new story of the family’s collective future outlook perspective. Both booster sessions will include guided-reflection questions for the family members to discuss with a trained OFOV facilitator. The general content of the storyboard booster will be guided by findings from formative phase, but the specific content of storyboards (booster 1) will vary by individual, and the specific content of the self-produced video (booster 2) will vary by family.

### Statistical analysis plan

We will use a rigorous intention-to-treat (ITT) analytic approach to evaluate the intervention outcomes of the pilot CRCT conducted under Aim 2. In an ITT approach participants are included in the analysis as originally assigned, regardless of whether they actually receive the intervention or SOC [55]. The study allocation sequence will be determined by the statistician. The randomization procedure will be carried out by the research coordinator. First, we will compare baseline data to see if randomization resulted in equivalent groups. If we determine non-equivalence, then the non-equivalent variables will be accounted for in the final analyses using a difference in differences analytic technique where randomization is used to minimize any perception of bias in selection of families by investigators. We will determine the proportions of HIV testing at 3- and 6-month of follow-up assessments among family members with 95% confidence intervals (CI). To test the primary study hypotheses that the intervention will increase uptake of HIV testing and PrEP (for HIV-negative members) and attendance at medical care appointment and ARV use (among PLHIV) within six months of randomization, we will use generalized estimating equation (GEE) with binomial distribution [56, 57], followed by effect size calculation with relative risk (RR). As odds ratios (OR) may overestimate RR for common events like those in our study, we will use a modified GEE with Poisson distribution [56, 57], which has robust error variances to estimate the RR and 95% CI of the OFOV intervention compared to SOC. Additionally, a modified Poisson regression approach will also be used to compute the RR and 95% CI for the binary outcomes (yes/no)–HIV testing, PrEP use, “engaged in HIV care” and “on ARV treatment”. These outcomes can be dependent on the health care facility (HCF) environment, such that participants who use services from the same HCF may be corrected or “clustered.” To account for the potential post-randomization clustering effect in this CRCT, a generalized linear mixed effects model with a logit link function will be fitted [58], and this model includes both HCF cluster-level and family-level factors.

### Ethical considerations

#### Ethics approval

This study #155461–11 was approved by the institutional review board of the State University of New York (SUNY) Downstate Health Sciences University Institutional Review Board (IRB) on April 16, 2020.

#### Process of consent

We will obtain informed consent from each individual family member prior to study enrollment. In addition, we will ensure that all participants know that their participation is completely voluntary and that they can withdraw at any time without repercussion. Informed consent will be documented by electronic signature on a touchscreen computer tablet and stored in the Research and Data Capture (REDCap) system on a secure SUNY Downstate Health Sciences University server. Prior to departing for the retreat, participants will also be asked to confirm that they still wish to participate.

#### Confidentiality and security of data

In order to prevent a confidentiality breach, all staff will be trained in how to protect participant information in a variety of circumstances (i.e., handling of paperwork, computer safety, file storage). The data will be entered into a new computer database that is kept private in several ways. Computers and tablets with study-related data are kept in locked office space, passwords are required to be used on all computers and tablets, and a separate password is required to use the database. Participants’ names and survey information will not be located in the same database. Participants will only be referred to by a unique alpha-numeric identifier.

#### Data storage and access

Participant’s names and survey information will not be located in the same database. The key linking the unique identifier to a person’s identity will be on a password protected computer, in a protected file, with limited access. All paper files will be kept in a locked file cabinet within the locked program office. Reports resulting from this study will describe aggregate data.

### Data and safety monitoring plan

The study PIs will review participant data bi-weekly, commencing 14 days after the first participant is enrolled in the study. The contact PI will chair the patient safety review team. The research study coordinator will prepare routine safety data reports for review by the research team. The study team will meet monthly or as needed throughout the study implementation to review safety data, as well as discuss and address any potential safety concerns. The study team will make all safety reporting to the SUNY Downstate Health Sciences University IRB within required deadlines. The study team will agree on the content and format of safety data reports prior to study implementation. A data safety monitoring board will not be providing oversight to this study; however, the SUNY Downstate Health Sciences University IRB and SUNY Research Foundation may review aggregate or individual-level safety data. In addition to the routine safety data reviews, the study team will convene on an ad hoc basis to make decisions regarding the handling of any significant safety concerns. If necessary, individuals external to the study team who represent expertise in the fields of community-based research, biostatistics, or medical ethics may be invited to join the safety review. A recommendation to stop the study may be made by the study team at any such time that the team agrees an unacceptable type and/or frequency of adverse events has been observed. In the unlikely event that the study team has serious safety concerns that lead to a decision to permanently discontinue the study for all participants and stop accrual into the study, the contact PI will immediately notify the SUNY Downstate Health Sciences University IRB and the SUNY Research Foundation. NIH will be notified of all safety concerns as required.

### Reporting of adverse events

This protocol presents minimal risks to the participants, and *Unanticipated Problems Involving Risks to Subjects or Others*, including adverse events, are not anticipated. In the unlikely event that such events occur, they will be reported immediately to the SUNY Downstate Health Sciences University IRB (if possible), followed by a written report within 5 calendar days of a PI becoming aware of the event. The investigator will apprise fellow investigators and study personnel of all *Unanticipated Problems Involving Risks to Subjects or Others* and adverse events that occur during the conduct of this research project as well as discuss it at monthly data and safety monitoring meetings.

### Dissemination plan

Research resources generated with funds from this grant will be freely distributed, as available, to qualified academic investigators for non-commercial research. The study PIs will adhere to the NIH Grants Policy on Sharing of Unique Research Resources including the “Sharing of Biomedical Research Resources: Principles and Guidelines for Recipients of NIH Grants and Contracts” issued in December 1999. The study PIs will also take responsibility for the following activities in support of public dissemination: (1) register the awarded study with clinicaltrials.gov within 21 calendar days of enrolling the first participant, (2) submit the study’s final results to clinicaltrials.gov within 1 year of the study’s primary completion date, (3) disseminate study findings (including intervention outcomes, study adaptation, and local community engagement) in peer-reviewed scientific journals and abstract presentations at scientific conferences, and (4) develop a community-centered fact sheet and study presentation to disseminate to local stakeholders.

### Protocol amendments

Any amendments to the study protocol will be submitted to the SUNY Downstate Health Sciences University IRB for review and approval prior to implementation. Changes that require IRB modification of study procedures may result in updated language in the consent forms. IRB modifications that retroactively affect participants may require contacting and re-consenting these individuals.

## Discussion

The HBC is a thriving community within the larger Black and Latinx LGBT communities. The HBC is organized to meet the needs of its members for social solidarity and mentoring in a society largely hostile to diversity in sexual and gender expression. This study investigates an assets-based approach to well-being and HIV prevention in the HBC. We have proposed to modify a skills-building and HIV prevention best-evidence, group-level intervention for HIV- negative Black MSM (3MV), into a family-based intervention (OFOV), to focus on asset-building for both HIV-negative Black MSM and Black MSM living with HIV within HBC families. At the end of the trial, we will utilize the data and findings to conduct a larger multi-level effectiveness trial of OFOV.

Findings from this study will aid in the development of future large-scale, culturally-tailored, status-neutral HIV prevention interventions that play key roles in boosting socioecological assets while buffering against the negative impacts of intersectional stigma. Such boosts in assets and reductions in intersectional stigma can facilitate improved engagement in HIV prevention and treatment (e.g., HIV testing and ART adherence). The importance of asset-building to the facilitation of resilience as a part of the domestic HIV response is recognized as a critical missing component that is welcomed by key national stakeholders in the HBC. Based on findings from the pilot CRCT, we will finalize a protocol for a full CRCT to conduct a definitive test of the efficacy of *OFOV* to improve HIV testing, PrEP use, care engagement, and ARV treatment.

## Supporting information

S1 File (DOCX)

## List of abbreviations

3MV: Many Men Many Voices
ADAPT-ITT: Assessment, Decisions, Administration, Production, Topic Experts, Integration, Training and Testing Model
AIDS: acquired immunodeficiency syndrome
ARV: antiretroviral
CAI: condomless anal intercourse
CDC: Centers for Disease Control and Prevention
CI: confidence intervals
Co-I: Co-Investigator
CRCT: cluster randomized controlled trial
DBS: dry blood spot
FGD: focus group discussions
HBC: house ball community
HCF: health care facility
HIV: human immunodeficiency virus
LGBT: lesbian, gay, bisexual, and transgender
MSM: men who have sex with men
NIH: National Institute of Health–United States
NYC: New York City
OFOV: Our Family Our Voices
OR: odds ratio
PLHIV: people living with HIV
PI: principal investigator
PrEP: pre-exposure prophylaxis
READY: Rochester Evaluation of Asset Development for Youth
REDCap: Research Electronic Data Capture
RR: relative risk
RCT: randomized controlled trial
STI: sexually transmitted infection
SOC: standard of care
SUNY: State University of New York
