# Supplementary material for: Leveraging family-based assets for Black men who have sex with men in House Ball Communities: Protocol for a cluster randomized controlled trial
Source: PLoS One. 2023 Sep 8;18(9):e0289681. doi: 10.1371/journal.pone.0289681 (PMC10490903; doi:10.1371/journal.pone.0289681)
Supplement: S1 File — (DOCX) [file pone.0289681.s001.docx]

**3.1 SIGNIFICANCE**

**HIV continues to disproportionately affect Black communities in the US.** Current epidemiologic data indicate that Blacks represent 45% of all new HIV infections, while representing 14% of the U.S. population.^1^ Most new HIV infections among Blacks have been observed in men who have sex with men (MSM). The estimated range of MSM representation in new HIV infections among Black males is 63%-66%^22^; the distribution of new HIV infections among Black MSM is increasing, with those younger than 25 years of age accounting for over half of the new infections via condomless anal intercourse (CAI) with other Black males (homophily)^1^; however, likelihood of engagement in HIV risk is influenced by a variety of socioecological factors.^23-29^

**Socioecological factors structure vulnerability to HIV infection risk for many Black MSM.**^29-32^ Some Black MSM seek refuge from environments that disaffirmed and/or attempted to dismantle essential aspects of their personhood, including their gender expression(s) and sexualities.^7,33^ Nonetheless, exposure to disaffirming environments has been reported to have residual negative psychosocial impacts.^34-36^ In our previous work, we found that early-life (before age 18) exposure to environments that disaffirmed same-sex behaviors was associated with increased number of sex partners and HIV infection in adulthood.^37^ In another study,^38^, we also found that financial crisis was associated with >2 male sexual partners in the past 6 months (OR=1.65; 95%CI 1.18–2.29), recent conviction was associated with sexually transmitted infection (STI) at 6-months (OR=3.97; 95%CI 1.58–9.94), and unstable housing was associated with an STI at 12-months (OR=1.71; 95%CI 1.02 = 2.86). The absence of “behavioral risk factors” does not confer protection against other economic and social hardships that still exert influence on HIV outcomes for Black MSM. It is therefore necessary to identify and strengthen assets to support resilience to hardships that can undermine the effects of HIV interventions.

**The House Ball Community (HBC)**—**a national network of Black LGBT kinship commitments (families)** provide caring, affirmation and survival skills-building for its members.^33^ It is a thriving community within the larger Black and Latinx lesbian, gay, bisexual and transgender persons (LGBT) communities. The HBC is organized to meet the needs of its members for social solidarity and mentoring in a society largely hostile to sexual and gender expression differences. Its earliest recorded origins date back as far as the 1920s during the Harlem Renaissance, although some of its artistic form was popularized in the 1990s by the song, Vogue—performed by Madonna. More recently, the stories of the community are highlighted in the FX TV-series “Pose”. The term “houses” refers to the actual familial networks, which are generally not localized in any one place or residence. The “ballroom” or “balls” are the common terms for the competitive dance and performance events which occur at regular intervals throughout the year and constitute an event circuit.^39^ Each House structure has a “father” and a “mother”, who assume the traditional biological family roles regardless of gender, sex or age; and act as surrogate parents to the House “children”, primarily LGBT adolescents, young adults and/or persons who have less experience and tenure within the HBC. House families can be modeled after a range of organizational structures including clubs and fraternities. Generally, they provide a safe haven and source of support for its members, LGBT youth of color, who have been rejected by their nuclear or biological families or other social institutions. The roles of a House parent range from serving as principal organizer, financial supporter, talent agent, health navigator, historian, production designer, special event planner and guidance counselor. An important role of Houses is to organize and compete in Ball events.^7^

The HBC population size is estimated to be approximately 8,000 nationwide, with the largest concentration in New York City.^39^ Despite the emerging visibility and celebration of the HBC’s contributions to culture in the Black LGBT communities; alarming disparities in HIV persist. Previous studies with HBCs identified HIV prevalence as high as 20%^40^ as well as high prevalence of stigma and stressful life events.^40,41^ Moreover, Black HBC members were more likely than non-Black Latinx members to be unaware of their HIV status (83% vs. 33%, p<.001).^41^

**To optimize HIV prevention outcomes for Black MSM, we must go beyond identifying and reducing risk factors towards simultaneously identifying and increasing assets** that are present in their lives. Although we don’t have data on assets among Black LGBT youth, the currently available evidence indicates that, in general, LGBT youth have fewer assets than heterosexual youth. In a population-based survey^21^ of assets among youth in grades 6-12, LGBT youth reported fewer assets than non-LGBT youth in the four family centered domains (see Table 1). The one exception was in the domain of “Constructive use of time”, wherein a greater percentage of LGBT youth reported being engaged in creative activities. Although the data in Table 1 do not show comparisons by race, there is already a substantial literature indicating that LGBT youth of color have worse outcomes compared to the general population of LGBT youth.^42-44^

**Table 1.** **Assets among LGBT Youth compared to non-LGBT Youth^45^**

| **Domain** | **Sample Assets** | **Non-LGBT (*n*=110,665)** | **LGB (*n*=5,645)** | **Trans**  **(n=722)** |
| --- | --- | --- | --- | --- |
| Support | Family Support | 75% | 55% | 49% |
|  | Positive Family Communication | 33% | 21% | 19% |
| Empowerment | Safety | 54% | 38% | 39% |
|  | Community Values Youth | 25% | 12% | 12% |
| Boundaries and Expectations | Family Boundaries | 47% | 37% | 32% |
|  | Adult Role Models | 33% | 21% | 16% |
| Constructive Use of Time | Youth programs | 66% | 55% | 58% |
|  | Creative activities | 19% | 27% | 29% |

**To date, there are no known group-level HIV behavioral risk-reduction interventions that also incorporate building family-based socioecological assets to optimize prevention and treatment outcomes for Black MSM.** Our team conducted a critical review^46^ of literature and only found five studies^10,47-50^ included content that identified socioecological assets to promote sexual risk reduction. Only two of the interventions that included socioecological asset promotion were targeted specifically to Black MSM: 3MV^10^ and d-up!.^47^ 3MV is a CDC-designated best-evidence group-level, peer-led intervention that addresses knowledge, attitudinal and behavioral factors influencing the HIV risk of Black MSM in the US. In an RCT, 3MV participants (relative to comparison participants), reported significantly more HIV testing and fewer episodes of CAI with casual male partners. One activity in 3MV includes a component that highlights how community interconnections were an asset that helped to successfully sustain Black communities during difficult periods throughout their histories in the Americas. Nonetheless, this specific asset is only identified and discussed in the intervention for illustrative purposes regarding historical resilience in Black communities. Asset-building is neither a core element of 3MV nor a component of its transtheoretical model and social cognitive theory foundations. In other words, there is neither systematic identification of what assets exist among the group of men who attend 3MV nor any effort at organizing the men to leverage assets to facilitate prevention outcomes within their group. Our review also found that two other interventions built on the cultural concept of familismo to promote inclusion of women as peer leaders to Latino MSM^49^ and family unity as means to generate social support for Latino gay-identified males;^48^ however, no data were presented indicating the percentage of their samples that identified as Black. We address this critical gap by incorporating an asset-building framework (See 3.2).

**The existing evidence-base for HIV interventions tailored for the HBC is extremely limited.** There is only one known HIV intervention tailored for the HBC. The *POSSE* intervention was a successful use of the peer-opinion leader model to adapt *the d-up! Defend Yourself! Intervention^47^* for use with House Ball Communities.^51^ The intervention represents the current best-available evidence for HIV interventions for HBC; nonetheless, there are several important gaps that remain in the state of science that we will address through this proposed R34 application. First, *POSSE* is based on an evidence-based intervention that is solidly grounded in a risk-reduction framework; however, it is not concurrently grounded in a socioecological asset-building framework to promote resilience to other social factors—such as stigmas—that MSM will encounter even after their HIV behavioral risk factors have been minimized. This is consistent with *POSSE’s* null finding on change in HIV stigma over time, even though there were statistically significant reductions in number of male sex partners and frequency of condomless anal sex. Second, *POSSE* incorporates HBC members as opinion leaders; but it does not include the HBC family-unit as a focus of the intervention. The *POSSE* study involved groups of individuals who were HBC members, but not necessarily members of the same family unit. *POSSE* was determined to be feasible and acceptable, but the pilot trial did not include a comparison group; thus, preliminary estimates of intervention’s effect size with HBC participants remains inconclusive. Our work builds on Hosek’s groundbreaking scientific contributions^51-56^ in least two ways: (1) co-anchoring a risk-reduction intervention to an asset-building framework that promotes identifying activating and leveraging existing assets and (where indicated) building new assets that can optimize HIV prevention and care and (2) centering the family-unit as the target of the intervention.

**Internal and external assets are key influencing factors on health seeking behaviors among MSM.** Building internal social assets as part of family-based socioecological assets is critical to facilitating engagement in health promoting behaviors.^57-62^ External assets are relationship, supports and opportunities such as family support, positive peer influence, and creative activities. Internal assets are skills and personal values such as self-esteem, sense of purpose, positive identity, planning and decision-making capacities. Although it is important to have interventions that focus on reducing behavioral risk for HIV, it is important that MSM be able to build interpersonal-level (peer-group/family) and intrapersonal-level (individual) capacities to challenge stigma, recover from stigmatizing experiences and to use strategies to protect against internalization of stigma that may result from chronic exposures to stigmas and hardships in society, including health setting.^63,64^

**3.2 SCIENTIFIC PREMISE AND THEORETICAL FRAMEWORK**

This proposal is premised on scientific and programmatic evidence that Black MSM’s vulnerability to HIV is not simply a function of behavioral risk; but, is also influenced by socioecological factors.^38,65-67^ The effect of evidence-based HIV interventions that focus on behavioral risk-reduction can be enhanced by integrating components that simultaneously identify and activate assets. HIV prevention and care outcomes for Black MSM in the HBC can be optimized by leveraging the assets that exist within HBC families. Our approach to this study is based on the **ADAPT-ITT model** for modifying behavioral interventions^68,69^ We theorize that Our Family Our Voices (*OFOV)* will (1) optimize HIV prevention and care outcomes by integrating post-intervention booster sessions to amplify the intervention’s influence on motivation and behavioral skills, and (2) build family assets that buffer against the potentially nullifying effects of intersectional stigma and other hardships—thereby facilitating the effect of the behavioral intervention to produce the primary outcomes (see Figure 1).^13^ We posit that combining risk-reduction with asset-building will produce a synergistic effect that cannot be attained through either approach alone.^13^ Our combined approach is needed because risk factors and contextual factors act interdependently to impede HIV prevention and treatment; therefore, reducing risk factors—while necessary—is insufficient to produce substantial and sustained effect in the context of intersectional stigma.^70,71^ HBC families are selected as the main unit of intervention because, even if Black MSM are sufficiently motivated for HIV prevention or care engagement, the socioecological conditions in HBC families can have either a protagonist or antagonistic effect. HBC families are also chosen because of the power of within-family peers to both positively influence healthcare-seeking behavior and buffer against the negative influence of intersectional stigma.^72^


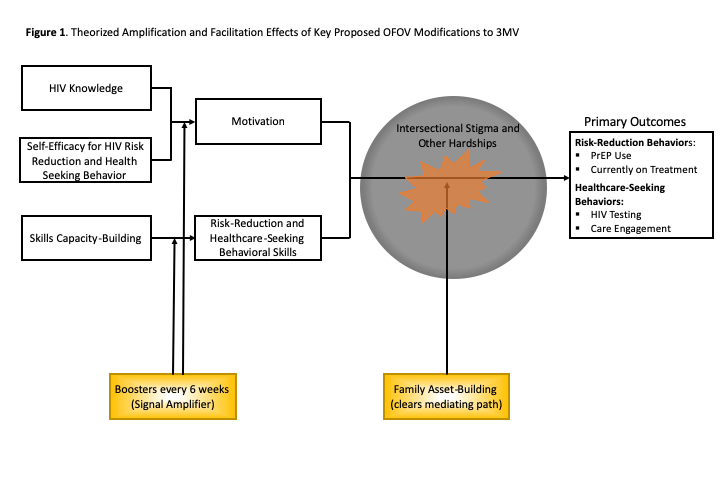


**Family-Based Asset Building Framework**

The asset building framework focuses on identifying external assets in four domains (i.e., support, empowerment, boundaries/expectations and constructive use of time) and internal assets in four domains (i.e., positive identity, positive values, social competencies and commitment to learning). The model includes 40 total assets and posits that increases in the number of assets is associated with increases in individual’s likelihood to show positive outcomes despite encountering adverse events.^19,21,73,74^ The goal of asset building is to identify as many of the assets as possible, acknowledging and encouraging those that are active and activating those that are dormant. Asset building complements the traditional risk-reduction approaches of identifying problems and reducing the likelihood of negative outcomes by identifying strengths and increasing the likelihood of positive outcomes.^73^ Using the inventory of 40 assets, we will be able determine which ones are common within HBC families and can be reinforced as well as which ones are less common but can be built in response to intervention. Further, the asset-building framework is ideally suited for families which are inherently diverse with regard to genders, levels of risk, and (HIV) health statuses. For example, asset-building does not rely on the existence of any level of “risk” that needs reducing. Finally, unlike ad hoc groups that are recruited to participate in the traditional 3MV intervention and then disperse, the model will build assets within families that exist within a broader network of HBC families—providing a nutrient-rich socioecological context of assets that can help to both facilitate and sustain the effects of the behavioral intervention.

**3.3 INNOVATION**

The proposed study offers multiple reinforcing innovations.

**(1) OFOV integrates risk-reduction + asset-building approaches to more precisely match the realities of life for Black MSM.** To date, most interventions to improve the prevention and/or treatment cascade have focused on the identification and elimination of “problems” and attended to a single socio-ecological level.^75^ The proposed study will use an existing intervention focused on reducing individuals’ risk behaviors and modify it by integrating a framework to build family-level assets—in an **integrated package**—that support the persistence of a risk reduction effect despite post-intervention exposure to life’s adversities. *OFOV* represents a departure from traditional HIV prevention designations of “high-risk groups” and embraces a more inclusive approach that allows participants to come together around shared family goals and values.

**(2) OFOV takes a family-based approach that facilitates an environment of resilience** among MSM to buffer psychological effects of stigma, discrimination and various hardships that may be encountered in the future. The impetus for this project comes from a six-year collaboration between the Health & Education Alternatives for Teens (HEAT) Clinic at SUNY Downstate and HBC leaders who were interested in identifying tools for parents of houses to support family members who were experiencing distress from various social hardships. Historically, stigma-reduction components of interventions focused on reducing negative attitudes of perpetrating individuals and institutions.^76-78^ Few interventions have leveraged social assets and cohesion among MSM peers to support the development of agency and autonomy to improve self-worth and resilience. *OFOV* will counteract intersecting stigmas by facilitating a family environment that is a source of affirmational and emotional support—all of which are shown to reduce social isolation and increase preventive health seeking-behaviors.^79-83^

**(3) Advances scientific theory on multi-level HIV status-neutral interventions** by testing an *OFOV* model that specifies how a behavioral risk-reduction framework *and* an asset-based framework model interact together to produce synergistic effects. It is further innovative for its focus on families rather than singling out sub-groups of family members based on HIV status. Implementation-related advances include that PLHIV may benefit from not having to not openly disclose their HIV status to participate in an intervention with other members of their community. The family gets the same information for HIV treatment and HIV prevention (e.g., PrEP, U=U) that becomes a communal exchange of knowledge within the family that has the potential to shift family norms, transform perceptions and minimize stigma. Status-neutral strategies represents a next generation response to the epidemic by dismantling HIV stigma that is embedded in the structure of interventions.

- 1. **APPROACH**

**3.4.1 Study Design Overview.** We will determine the feasibility, acceptability and a preliminary estimate of effect size of an evidence-based HIV prevention intervention for Black MSM adapted for HIV status-neutral use with families in the HBC (called: *OFOV*). The eight-step ADAPT-ITT framework^17,18^ will guide our approach to enhancing the intervention’s content to incorporate an asset-building framework that leverages the strengths of the families. Aim 1 will encompass steps 1-7, leading to the combined multi-level intervention adapted to improve congruence with the realities of HBC family life. Aim 2 encompasses step 8, in which we will assess the feasibility and acceptability of conducting a cluster randomized controlled trial (CRCT) of *OFOV* with a standard of care wait-listed control condition (see section 3.6.1). We will use data collected from the CRCT to estimate the effect size of *OFOV* on **HIV testing and PrEP use** (primary outcome for HIV-negative participants), **HIV care engagement** and **currently on ARV treatment** (primary outcome for PLHIV), as well as **number o**f **family-based assets** and **number of sex partners** and **relative** **frequency of CAI** (status-neutral secondary outcomes). In deciding on the wait-listed controlled CRCT we considered other designs such as factorial designs and a classic RCT design with time and attention-matched controls. We do not yet seek to isolate which component of the intervention has the greatest effect on our outcomes; thus, a factorial design was not scientifically justified for our proposed study. Additionally, we decided to forgo a structurally equivalent control group given the evidence of 3MV’s efficacy in risk reduction and increasing HIV testing.

**3.4.2 Investigative Team**. Our team brings expertise in (1) community-engagement with HBCs, (2) clinical and research expertise with Black MSM, (3) program implementation of 3MV, (4) ethnographic research and (5) RCTs. This multidisciplinary team is uniquely qualified to carry out this research, led by partnering PIs with complementary disciplinary, clinical, research and intervention experience with Black MSM and HBCs. This includes many years of experience recruiting and engaging families in HBCs into intervention programs and clinical services. **Dr.** **Jeffrey Birnbaum** (Contact **MPI**) is a primary care physician and who has over 15 years of experience in the provision of clinical care, community-based programming and engagement in research with members of the HBC in New York City. **Dr.** **LaRon E. Nelson** (**MPI**) is a family nurse practitioner with has 15 years of HIV research experience grounded in 17 years of clinical nursing practice. He was a member of the team that evaluated 3MV and has experience in multiple federally funded HIV behavioral intervention trials with Black MSM.^14,37,38,84,85^ **Dr. Sabina Hirshfield** (Co-I) is an expert in conducting HIV prevention intervention trials with racially/ethnically diverse MSM and has reached over 75,000 participants during her research career.^86-93^ **Dr.** **Marlon M. Bailey** (Co-I) is an HBC member, a cultural ethnographer and the world’s leading scholar on the construction of families and the function of performance within HBCs.^7,16^ **Dr. Han-Zhu Qian**, (Co-I) is an epidemiologist with research focus on HIV prevention studies among MSM and a biostatistician with substantial experience in RCT study design and data analysis. **Mr.** **Michael Roberson** (**consultant**) is one of the initial developers of 3MV and a leader in the HBC. He has collaborated with MPI Birnbaum on the programmatic implementation of 3MV at HEAT. He is the father of an HBC family and is a key social influencer on HBC visibility in popular culture. He serves as an FX Network consultant to the writing team of “Pose” and provides training and capacity building to the Pose technical crew on gender, sexuality and ethno-racial sensitive approaches for working with HBC cast members. **Dr. Charlene Sinclair** (consultant) is a leading scholar on leadership and community organizing and has experience working with HBC on the use of community organizing principles to attain health and social equity. **Mr. Serette King** (consultant) is an expert in the development and implementation of programs to that address the health and psychosocial needs of young Black MSM in HBCs by reaching this population in non-mainstream settings and identifying opportunities to facilitate linkage to health/human services.

**3.4.3 Study Setting.** The study will be conducted in the New York City (NYC) metro area, which is the geographic area with the highest concentration of individuals in the HBC. The pilot study described in Aim 2 will be conducted with six HBC families that have never been previously exposed to the original 3MV intervention.

**3.4.4** **Preliminary Studies -** **Evidence of Program Experience, Capacity and Engagement with HBCs**

**3.4.4A. HEAT’s capacity to successfully engage HBC members in 3MV.** HEAT (see Box 1), under the direction of MPI Birnbaum, has implemented 11 cycles of 3MV from January 2017 through June 2019, successfully enrolling 174 HBC members for whom 153 (87.9%) completed the intervention for all sessions. HEAT conducted these sessions in both local (in NYC) and out-of-town retreat formats. The local format is conducted over a 3-day period where participants are dismissed at the end of each day and asked to return to complete the intervention. The out-of-town retreat format is also conducted over a 3-day period; however, all participants are transported by bus for the intervention that is held on-location at an all-inclusive site that includes lodging and meals. Our 3MV 3-day retention is 68% for the local format and 100% for the retreat format. In 2017, HEAT began “family-based” recruitment to fill some of the cycle cohorts of 3MV, making these cohorts unique in that cohort members all had pre-existing social and HBC-kinship ties through their house membership. During these house-based cohort cycles, elements of house membership as part of the participants’ identities and the positive role house/HBC membership played in their lives were topics which were informally integrated into 3MV.

**Box 1. Community Partners**: HEAT Clinic **(HEAT)**, Federation of Ballroom Houses **(The Federation),** House Lives Matter **(HLM) and** the Kiki Coalition **(The Coalition).** The investigator team is partnered with grassroots community-based organizations (**gCBOs**) who were active contributors of feedback and ideas that informed the development of the current proposal and who support the research proposed. The **HEAT Clinic** at SUNY Downstate is a hub of excellence for HBC-centered primary care. The **Federation, HLM** and the **Coalition** are gCBOs that work to advance the social, and economic interests of the HBC. The gCBO leaders have solid, long-standing relationships with the MPIs and have collaborated on previous HBC-focused programs and evaluations. All of the gCBOs have successful track-records of respectfully and humanely engaging, recruiting and retaining HBC members.

**3.4.4B. Capacity to engage HBC members in clinical services**. During the same period of 2017 to 2019, HEAT, in collaboration with **The Federation** and **The Coalition (**see Box 1**)**, engaged 57 HBC members living with HIV into HIV primary care and 33 in PrEP medical services. Many of the HBC members entering clinical care at HEAT are referred through or enter care because of HEAT’s HBC engagement. HEAT’s engagement with the HBC extends past intervention groups such as 3MV. HEAT additionally hosts its own HBC events including balls and smaller house-based events such as “house” parties, game night, movie events and others where HIV and STI screening are offered outside the clinic. Through entry into care at HEAT, HBC members have access to HIV 4^th^ generation Ag/Ab testing, HIV viral load testing, screening/testing/treatment for syphilis, gonorrhea and chlamydia, and prescription of ARV and PrEP. This ability to engage HBC members both in a clinical capacity as well as in the community is a key strength of HEAT as a partner in the proposed study.

**3.4.4C. Pilot of 3MV to include HBC members living with HIV.** In 2010, MPI Birnbaum and Mr. Roberson adapted 3MV for use with HBC youth living with HIV (NYC AIDS Fund, October 2010).
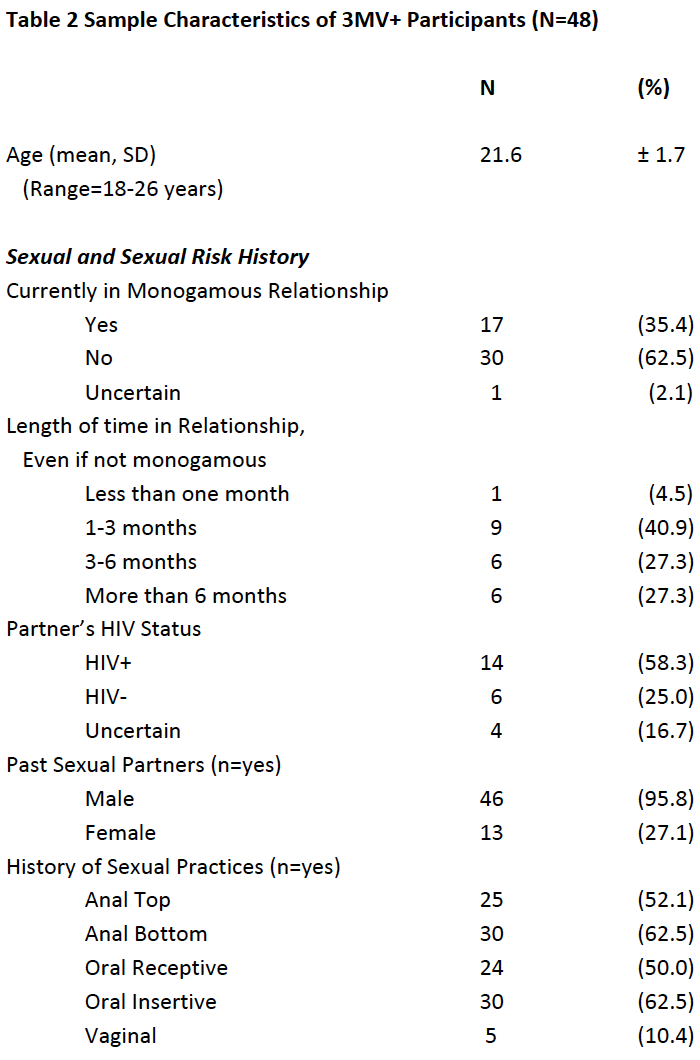
We worked with HBC leaders to adapt both the 3MV curriculum and evaluation tools by including increased attention to HIV stigma and identity development for HIV+ youth, the importance of HIV prevention behaviors for those already living with HIV, and issues of HIV disclosure in sexual relationships. The team also adapted the intervention language to make it more relevant for HBC youth. HEAT conducted four pilots of the adapted intervention with 48 HBC members recruited from HEAT and gCBO partners (see Table 2). Each pilot included 15-20 HBC youth (ages 17-23). Fiscal constraints in the programmatic grant used to support this particular project necessitated that we conduct the adapted 3MV over six weekly sessions; however, we noted that this format had a negative impact on retention (~70%) compared to the weekend retreat format which demonstrated far superior retention (100%). Participants received knowledge, attitudes, behavior, and belief assessments at 2-weeks, 3-months, and 6-months post intervention. Results from the pilot demonstrated: 1) a 33% increase in engagement in HIV care among project participants, including improved appointment show rates and increases uptake of ARV medication; 2) significant increases in condom use for anal sex (p < .05); at 3- and 6-months and 3) the creation of a social network among study participants. These data demonstrate the feasibility and acceptability of incorporating content for PLHIV into the 3MV intervention as well as our capacity for longitudinal follow-up and data collection. The 3MV intervention manual will be rigorously refined to focus on identifying and building assets within HBC families and addressing other stigmas that intersect with HIV stigma.

**3.4.4D. 3MV Focus Group for HBC Cohort Members.** In 2018, **MPI Nelson** conducted a 3MV Focus Group with 8 participants who participated in HEAT cycles of 3MV with a house-based cohort. A key finding of the focus group discussions is that when 3MV is custom-tailored for individuals within the HBC, some individuals expressed having negative attitudes towards the HBC prior to the intervention. However, the experience of the intervention, which highlighted the history and assets of ballroom shifted their perceptions to more positive attitudes. Another key finding was that 3MV linked people together who are connected via their houses, but who were not previously connected to each other as a source of social support because they were not necessarily a member of the same “family”. A major recommendation of the focus group was that 3MV should not be just a “one-time” intervention but that its infrastructure should be expanded to sustain intervention effects. This feedback profoundly influenced our decision to develop *OFOV* to target the HBC family-unit, because it is an existing infrastructure that has built-in social continuity of engagement, and to include multiple booster sessions.

**AIM 1: CONDUCT FORMATIVE RESEARCH TO IDENTITY KEY MODIFICATIONS TO 3MV INTERVENTION MANUAL AND IMPLEMENTATION PROTOCOL**

**3.5 FORMATIVE PHASE.** The ultimate objective of Aim 1 is to adapt the 3MV intervention to the OFOV intervention. A schematic overview of the formative phase of Aim 1 is depicted in **Figure 2.**


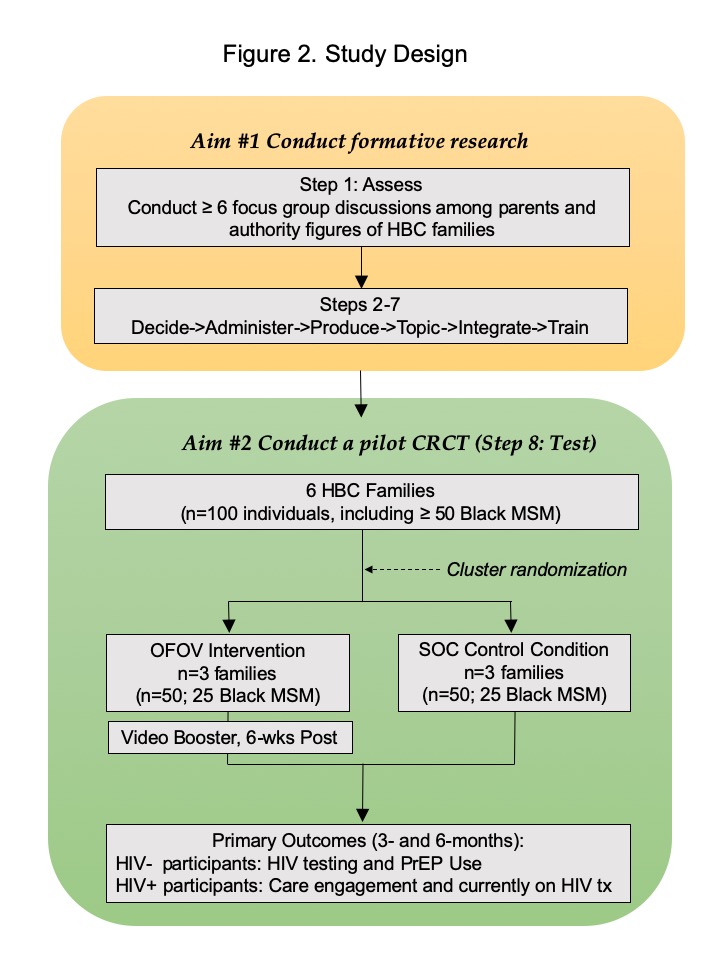
**3.5.1 Step 1 (Assess):** We will conduct focus group discussions (**FGDs**) with parents and authority figures of HBC families to inform the adaptation of the 3MV interventions by deepening our understanding of the range assets in the external (i.e., support, empowerment, boundaries/expectations and constructive use of time) and internal (i.e., positive identity, positive values, social competencies and commitment to learning) domains. The focus groups will also explore their observations of the various stigmas and hardships faced by family members, how these stigmas intersect, and their views on what are best combinations of assets to buffer against the negative effects of intersectional stigma on HIV testing, PrEP use, linkage to care and ARV use. FGDs were chosen because of their suitability for sexual health studies^83,94-96^ and to also capitalize on the type of spontaneous conversation interactions that occur. Re-creating the social dynamics among parents and authority figures is critical to understanding possibilities for assets-building within the social organization of HBC families

We will qualitatively:

1. Characterize the external and internal assets that exist within families, including patterns of commonalities and differences between families.
2. Identify sources of exposures to intersectional stigma among HBC family members.
3. Explore the various ways that intersectional stigma influences health goals in HBC families.
4. Explore content for inclusion in video booster sessions to amplify post-*OFOV* knowledge and skills and counteract intersectional stigma to facilitate the primary study outcomes (see Figure 1).

The data generated in this step will inform how HBC family dynamics, social realities, and assets are addressed in the *OFOV* intervention components.

**3.5.1A Sample Size and Sampling Strategy**. We will conduct 6 FGDs with parents of HBC families. We anticipate that 6-8 parents or parental authority figures will participate in each focus group. Thus, the estimated sample size is between 24-48 parents. Based on our many years of experience working within the house ball community and our geographic concentration in the New York City metro area (inclusive of northern New Jersey and southern Connecticut), we do not expect a wide variation in responses between the focus groups; therefore, we expect that we will reach data saturation by the fourth focus group. Nonetheless, we are prepared to conduct more FGDs if our qualitative analysis reveals that new codes are still emerging even after completing 6 FGDs.

We will conduct a chain-referral method to recruit the sample via **The** **Federation**, ***HLM*** and **The Coalition** (our HBC gCBO partners). Chain-referral was chosen based on the efficiency of tapping into an existing extensive network in which trusted relationships with our community partners can facilitate interest in participating in the study. This process involves recruiting parents who are known to **The Federation, HLM**, and **The Coalition**. They will be asked to invite other parents (whom they consider to be their peers) from their social network to participate in a focus group, as well as members of their families whom they deem authority figures to their house children. As described in section 3.4.4 above, this team has a successful history of recruitment in the HBC. Although we are not employing a purposive sampling strategy, we are conscious of the insidiousness of transphobia; therefore, we will monitor patterns of the participation of transgender-identified individuals and may target recruitment to transgender individuals if we find that they are under-represented in the focus groups.

**3.5.1B. Inclusion criteria for current HBC members**. Individuals are eligible to participate in the FGDs if they are at least 18 years old, are the publicly recognized parents or authority-figures of an HBC family, and their immediate house family-unit has at least twelve members. Individuals will be excluded if:

- Their HBC family has been established for less than 12 months
- The individual lives outside of the New York City metropolitan area

**3.5.1C Data Collection.** FGDs will be conducted by trained research staff from SUNY Downstate who have prior experience in qualitative research methods within Black LGBT communities. We make efforts to employ individuals from the HBC as a community capacity-building and investment tactic. All qualitative data will be digitally recorded and transcribed verbatim. The research project staff will be responsible for the transcription. All transcripts will be verified by two research project staff. After interviews are transcribed and verified for accuracy, recordings will be encrypted and uploaded to a secure cloud-based server at SUNY Downstate.

**3.5.1D Data Analysis**. We will conduct qualitative content analysis, a procedural approach in which data are categorized and reviewed iteratively to generate conclusions based on both explicit and implicit meaning in the text.^97^ We will use NVivo to manage the qualitative data. Nelson and Bailey will lead the qualitative analysis with assistance from **Birnbaum** and **Sinclair.** Dr. Sinclair will not have any direct access to the qualitative data. During the preparation phase, **Nelson** and **Bailey** will read the FGD transcripts independently several times to become immersed in the data. Open coding will be used to identify nodes. Open coding will continue until no new nodes are identified. Through an iterative approach, node groups will then be formulated into meaningful categories and sub-categories. Quotes that represent the various categories and sub-categories will be selected and used to illustrate the content of the FGDs. Findings will be discussed among all the investigators and HBC gCBO partners to ensure a balanced and accurate representation of assets and stigmas and how these findings can best be represented and addressed through activities in the adapted intervention. We will also use the guide in the development of storylines and scripts for the video booster sessions will be directed/produced by **Michael Roberson** and co-produced by **Serette King**. Mr. King will not have any direct access to project data.

**3.5.2 Step 2 (Decide).** Guided by the formative research results, the team will meet to discuss what specific intervention activities should be modified to incorporate family-based assets. We will conduct an updated scoping review of research literature to identify innovative evidence-based options to enhance focus on assets to facilitate resilience, with special attention to technology and social media options. This will include a review of the current intervention activities and discussion of how they can be refined to include identifying and building assets against intersectional stigma and hardships. In consultation with **The Federation, HLM** and **The Coalition**, we will incorporate changes into the curriculum, identify gaps/errors in logic and make corrections in preparation to conduct a workshop of the intervention with a reference group of HBC families.

**3.5.3 Step 3 (Administer):** After initial modifications, we will conduct a facilitated simulation workshop of the *Our Family Our Voices* intervention activities with two HBC families who previously received the original 3MV. We will conduct separate workshops (versus one joint workshop) because it is consistent with the concept that the intervention is targeted to the family-level and we want to get feedback on its performance within a dynamic that is closest to the one we expect to target in the pilot RCT phase. We will produce a preliminary revised version of the original intervention manual before the workshop and conduct a series of facilitated simulation exercises with role-play over a 3-day period. Participants will work through each of the intervention components, focusing on the family asset-building content and reviewing our approach to “connecting the dots” that these assets are socioecological resources that can buffer against the negative impact of intersectional stigma.

**3.5.4. Step 4 (Produce):** Based on the feedback from the simulation workshop participants and our observations of their engagement with the activities, we will determine what content and approaches to incorporate into the *OFOV* intervention manual and which ones to edit or forego. This includes making final decisions regarding the final storylines and messages for the video boosters. **Sabina Hirshfield** (co-investigator) is an expert in the development of video vignettes for use as components of HIV-related intervention with MSM. ^98-100^**Dr. Hirshfield** will work with **Michael Roberson** and **Serette King** on the development and production of vignettes as brief stand-alone videos for viewing every 6-weeks (weeks 6, 12, 18, 24) between 3- and 6-month follow-up. We will give priority consideration to retaining components that have the highest consistency with scientific evidence on HIV risk-reduction and asset-building as well as HBC cultural relevance based on input of **HBC gCBO** partners.

**3.5.5 Step 5 (Topic):** We will engage topic experts to review the adapted manual and provide feedback on its congruence with the original intervention and local sociocultural relevance. A copy of the adapted intervention manual will be provided to **Marlon M. Bailey** (co-investigator) is the world’s foremost authority on House Ball culture and factors that influence HIV prevention and care—will review the manual to ensure that it has resonance with ballroom and to avoid contrived product that does not elicit natural authentic connection with members of the HBC. **Michael Roberson, Charlene Sinclair** and **Serette King** (consultants) will review the intervention for cultural resonance as well as to ensure that it maintains fidelity to internal logic of the 3MV intervention on which it is based. Dr. Sinclair and Mr. King’s role as paid community consultants is essential to inform the acceptability of the intervention with the focus population but will not have direct contact with collected data. **Our gCBO** partners will give feedback that is key to the local cultural relevance and implementation of OFOV. Letters of support from the topic experts and HBC gCBO partners are included.

**3.5.6 Steps 6 (Integrate) and 7 (Train):** In this step, we will take feedback provided by the topic experts and summarize it in a report that will be distributed to the entire investigative team, including our key collaborators. We will discuss the feedback and provide follow-up clarifying questions to the topic experts, as necessary, before making final revisions to the adapted intervention manual. We will also produce a training manual to standardize training and permit future replication of the intervention in other settings (e.g., HBC families living in southern of United States). We will provide comprehensive training to facilitators whose roles are dedicated to delivering the intervention **(see 3.6.7A, Training of Interventionists)**. We will develop the training to be multi-faceted to facilitate comprehension and retention of training concepts, using strategies that Drs. Birnbaum and Nelson^101^ have successfully deployed in other HIV prevention research projects. This will include conducting a joint (including with representatives from **The Federation, HLM,** and **The Coalition**), dry-run rehearsal of the protocol prior to implementation in the pilot CRCT with HBC families.

**AIM 2: DETERMINE THE FEASIBILITY AND ACCEPTABILITY OF THE MODIFIED INTERVENTION**

**MANUAL AND TRIAL PROTOCOL**

**3.6 RANDOMIZED CONTROLLED TRIAL PHASE**. An overview of the RCT phase of is depicted in **Figure 2.**

**3.6.1 Step 8 (Test).**

**Overview of Trial Design.** Using a cluster RCT, we will evaluate the feasibility and preliminary efficacy of *OFOV* compared to waitlist standard of care (SOC) control on uptake of HIV testing and PrEP use (for HIV-negative individuals) and linkage to care and current ARV uptake (for PLHIV) among 6 New York City HBC families over a 6-month period. SOC is defined as the currently available suite of prevention and care services in the NYC metropolitan area. The SOC control will not receive a group/family-based behavioral risk-reduction intervention. Since the HBC is relatively small and recruitment is limited to the NYC metropolitan area, we propose a cluster-randomized design to reduce the likelihood of contamination across families in the HBC. A limitation of randomizing by clusters is a loss of efficiency; thus, the samples size needed to obtain precision in a cluster-randomized trial is usually larger than when randomizing individual participants. Once a cluster is randomized to the treatment condition, it will remain in the assigned condition for the duration of the study. Using this study design, each cluster (family) in the experimental arm will receive both the OFOV intervention over a 2.5-day weekend retreat and video booster sessions every 6-weeks thereafter until the 6-month data collection point. Families in the experimental arm will be asked to follow-up for HIV testing and PrEP services (for HIV negative or status-unknown individuals) or linkage to treatment services (for PLHIV).

Our working hypotheses are that the *OFOV* intervention will increase uptake of HIV testing and PrEP use (for HIV-negative family members) and linkage to care and treatment engagement (for PLHIV who were not in care at baseline). We will measure frequency of HIV testing at 3- and 6-months post-intervention using a combination of online self-report survey and verification by through clinic medical records. We will seek to obtain medical records releases from participants to obtain data on HIV testing, PrEP prescription, engagement in care and ARV use. Our secondary hypotheses are that *OFOV* will increase the number of family-based assets, resilience and reduce number of sex partners and frequency of CAI for members who are not in mutually monogamous relationship with an HIV-negative or virally suppressed partner. We also measure intersectional stigma and asset-impact indicators at all time points (to characterize these in our sample) ^102,103^ Families in the waitlist SOC control arm have the option to participate in OFOV after 6-month data collection.

**3.6.2 Procedures**

**3.6.2A Recruitment and Sample****.** We will recruit six HBC families (n=100). See Human Subjects section *4.4. Statistical Design and Power* for sample size and power calculations. We will conduct recruitment using three methods. First, we will disseminate recruitment/marketing materials via **The Federation**, **HLM** and **The Coalition’s** online communication platforms. Potential participating families can contact the study office directly via text message or phone call in response to marketing materials. Second, we will recruit through receipt of referrals of interested families from **The Federation**, **HLM** and **The Coalition’s** programs and outreach activities. Research staff have extensive experience engaging MSM for research and program activities. Third, we will also rely on word-of-mouth as a mechanism through which families become aware of the study and contact us.

**3.6.2B Inclusion/Exclusion Criteria.** Eligibility for the study will be determined primarily at the family-level. At least 12 members of the same HBC family must be willing to participate together, of which at least half the participating members must be Black MSM. Participating family members must reside in the New York City metro area. Black MSM participants must be at least 18 years old. Dr. Birnbaum, who is a pediatric HIV specialist, believes that the social risk of a confidentiality breach to the biological family is heightened for youth under the legal age of emancipation. Furthermore, we could not justify the risk of enrolling people younger than 18 since our research aim can reasonably be addressed without their inclusion. In addition, Black MSM must also currently self-identify as cisgender or transgender man, report anal sex with another man at least once within the previous six months (note: other HBC family members can identify as any gender).

**3.6.2C Informed Consent.** We will obtain informed consent for each individual family-member prior to study enrollment. HEAT project staff will be responsible for obtaining and ensuring informed consent and will fully explain the study to potential participants and answer all questions regarding what study participation entails. We will ensure that all participants know that their participation is completely voluntary and that they can withdraw at any time, without repercussion. After consenting, participants be given a link to electronically complete the baseline survey on their smartphone, tablet, or desktop computer. Informed consent will be documented by electronic signature on touchscreen computer tablet and stored in research and data capture (REDCap) system. Prior to departure on the retreat, participants will also be asked again to confirm that they still wish to participate.

**3.6.2D Enrollment and Randomization.** Consented individuals will be enrolled in the study consecutively. Given the relatively small number of facilities per arm (N = 3), a covariate-constrained optimization technique (e.g., matching) will be used to randomize intervention assignment and minimize imbalance in the two arms.^104^ This randomization method will be examined in this pilot for consideration of using in future full RCT.

**3.6.3 OFOV Intervention Implementation**

**3.6.3A Retreat Procedures**. Families who are assigned to the intervention arm will be given dates for the weekend *OFOV* retreats they would attend at temporarily rented facilities. Prior to attending *OFOV*, participants will attend an orientation meeting at HEAT to obtain information about the logistics and format of the retreat and to establish ground rules for appropriate behavior. Once participants arrive at the retreat, the *OFOV* intervention will be delivered in six consecutive 2- to 3-hour sessions. The original 3MV sessions content is summarized below (see Table 3). HIV Counseling & Testing staff from HEAT will participate as guest facilitators in one of the *OFOV* sessions and will use that opportunity to reach out to family members to visit their facility for HIV testing, PrEP, engagement in care or HIV treatment.

**3.6.3B Video Boosters.** Four self-contained, video booster session modules will be delivered every 6 weeks post *OFOV* intervention (approximately two boosters between 3 and 6 months). The video boosters are designed to be watched together as a family. The videos will include guided-reflection questions for the family members to discuss. The content of the video boosters will be developed in the formative phase **(see Section 3.5.4).** Based on **Dr. Hirshfield’s** previous work, potential topics of videos range from dealing with HIV status or STI disclosure, importance of consistently taking ARVs, and effective advocacy for family members’ health needs in healthcare settings. The videos will only be available to study participants via a secure URL and will not be able to be forwarded to friends or other participants (preventing cross contamination between arms). Each individual will receive the link to the video and may watch the video before or after the family convenes their group session.

**Table 3. Summary of Original 3MV Intervention, Sessions Topics and Length**

| **Intervention** | **Level** | **Modality** | **Sessions** | **Length (Mins)** |
| --- | --- | --- | --- | --- |
| Many Men, Many Voices /3MV adaptation for HBC-***Our Family Our Voices*** | Peer-Group and Family | Group Sessions using:  *Brainstorms*  *Role Play*  *Small group*  *activity*  *Paired work*  *Develop menu*  *of prevention*  *options*  *Action planning* | 1. MSM and dual identity | 140 |
|  |  |  | 2. HIV/STD prevention for MSM: the roles and risks for tops and bottoms | 210 |
|  |  |  | 3. HIV/STD risk assessment and prevention options | 110 |
|  |  |  | 4. Intentions to act and capacity for change | 170 |
|  |  |  | 5. Relationship issues: Partner selection, communication, and negotiation of roles | 140 |
|  |  |  | 6. Social support and problem solving to maintain change | 125 |
|  |  |  | 7. Building bridges and community | 130 |

**3.6.3C Selection and Training of Interventionists.** Each *OFOV* intervention retreat will be conducted by two facilitators from the HBC. We will identify and train 6 (4 primary and 2 back-up) intervention facilitators from the group of HBC members who have previously attended 3MV retreats. This pool will already have had the experience of undergoing the original intervention and will therefore be more sensitized to the topics and the potential anxieties and wariness of the intervention participants. The facilitators will first receive a two-day training on group facilitation skills in which they will increase their knowledge, confidence and fluidity in group facilitation. They will then be trained on *OFOV* as well as on current state of the science topics, such as U=U.

**3.6.4 Data Collection and Measures.**

**3.6.4A Data collection.** Participants will have the option of either receiving an electronic link to complete baseline, 3- and 6-month surveys or they can visit the HEAT clinic and complete the online survey using internet-enabled computer tablets. Survey data will be collected using research and data capture (ReDCap) software. All trial participants will receive the same surveys, with the exception that only the intervention arm will be assessed on implementation process measures (Tables 4). Participants will be given gift card incentives after the survey completion at baseline, 3 and 6 months for a card value of $50, $75 and $75, at those respective time points.

We will also collect data via medical record review. All participants will be asked to complete a medical record release authorization form that will allow us to request the following information from their medical provider(s): HIV testing in past 3 months, last prescription written for HIV PrEP or last ARV prescription written for treatment and last PrEP or HIV medical appointment kept.

**3.6.4B Demographic, Knowledge and Behavioral Factors**. We will assess important baseline characteristics for describing the sample including age, income, gender, education, ethnicity, relationship status, religion, sexual identity, sexual behavior, HIV knowledge, HIV testing readiness, HIV testing history. We will characterize the sample on intersectional stigma by creating a latent variable that is drawn from three scales that measures HIV stigma, same-sex stigma, and gender non-conforming stigma (see Table 4).

**3.6.4C Primary and Secondary Outcomes.** Our primary outcomes will be HIV testing and PrEP use (for HIV-negative or unknown members) or “In HIV care” and “currently on HIV treatment” (for PLHIV) within 6 months of randomization. For HIV-negative MSM we will also record the frequency of HIV testing over the prior 3-months, but we anticipate that few participants will take >1 test in this short period of time. A detailed summary of primary outcome measures is included in Table 3. Our secondary outcomes (see also Table 4) will be number of family-based assets, resilience, number of sexual partners, and relative frequency of CAI. We will also assess sense of community^105^ at all time-points. In our previous research with Black MSM we found that sense of community was associated with increased condom use for anal intercourse (*OR*=1.26, 95%CI 1.05,1.52; p<.05).^14^

**3.6.4D Implementation Outcomes.** We will assess feasibility and acceptability of *OFOV* using a combination of process measures (e.g., intervention completion rate) and validated scales (e.g., peer support evaluation inventory). Implementation outcomes measures are also summarized in Table 4.

**3.6.5 Data Analysis.** The intervention outcomes will be assessed using a rigorous intention-to-treat (ITT) approach where participants are included in the analysis as originally assigned, regardless of whether they actually receive the intervention or usual care.^111^ First, we will compare baseline data to see if randomization resulted in equivalent groups. If we determine non-equivalence, then the non-equivalent variables will be accounted for in the final analyses using a difference in differences analytic technique where randomization is used to minimize any perception of bias in selection of families by investigators. We will determine the proportions of HIV testing at 3- and 6-month of follow-up assessments among family members with 95% confidence intervals (CI). To test the primary study hypotheses that the intervention will increase uptake of HIV testing (for HIV negative members) and attendance at medical care appointment and ARV use (among PLHIV) within six months of randomization, we will use an uncorrected chi-square statistic, followed by effect size calculation with relative risk (RR). As odds ratio (OR) may overestimate RR for common events like in our study, we will use a modified Poisson approach to estimate RR of intervention relative to SOC and CI by using robust error variances.^112^ Additionally, a modified Poisson regression approach will also be used to compute the RR and 95% CI for the binary outcomes (yes/no) – HIV testing, PrEP use, “engaged in HIV care” and “on ARV treatment”. These outcomes can be dependent on the health care facility (HCF) environment, such that participants at the same HCF may be corrected or “clustered”. To account for the potential post-randomization clustering effect in this CRCT, a generalized linear mixed effects model with a logit link function will be fitted, and this model includes both HCF cluster-level and family-level factors. **See Human Subjects section 4.4. for Power Calculations.**

The likelihood of intervention contamination across the study groups could not be excluded, because some participants may not receive the respective interventions to which they are randomized, e.g., some members of the control group may end up in situations in which they receive some level of support from a HBC family in the intervention group. Therefore, we will also perform per-protocol analysis, which includes only those participants who strictly adhered to the protocol and report the results along with the results of the ITT analyses.

| **Table 4. Summary of RCT Phase Measures** | |
| --- | --- |
| **Construct** | **Sample Items and reliability** |
| **HIV Testing** (self-report and medical record verified) | Number of HIV tests in past 3-months |
| **PrEP Use** (self-report and medical record verified) | Is participant currently on antiretrovirals for HIV PrEP? |
| **In HIV Care** (self-report and medical record verified) | Has participant attended HIV medical appointment in past 3-months |
| **On HIV Treatment** (self-report and medical record) | Is participant currently on antiretrovirals for HIV treatment? |
| **Condomless anal intercourse** (past 3 months) | Relative frequency = # times condoms used during anal sex / total # episodes of anal sex |
| **Number of sexual partners** | Total number of sexual partners in the past 3 months |
| **Sense of Community Scale^105^** (8-items) | I feel like I am a member of my house family (α=0.87 - 0.92) |
| **Brief Resilience Scale^106^** (6-items) | I tend to bounce back quickly after hard times? (α=0.80-0.91) |
| **READY Tool** (24-items)^102,103^ | My family expects me to try my best. I ask my family questions to get ideas. (α=0.85) |
| **Asset Inventory (40-items)^21^** | I seek advice and counsel from my parents. My family has clear rules and consequences. |
| **Intersectional Stigma (Latent Variable)** |  |
| **HIV Stigma Scale^107^** | |
| *Enacted subscale (10-items)* | Has a healthcare worker not wanted to touch you because you have HIV? (α=0.92) |
| *Vicarious subscale (10-items)* | How often have you heard stories about someone with HIV being refused care? (α=0.88) |
| *Perceived subscale (10-items)* | In your community, how many people think a person with HIV is disgusting? (α=0.94) |
| *Internalized subscale (10-items)* | How often do you feel guilty about having HIV? (α=0.83) |
| **Same-sex stigma scale** ^108^(10-items) | How often have you lost a place to live for being homosexual? (α=0.75) |
| **Gender Non-Conformity Stigma Scale^109^** (10-items) | How often have you been called names because of your feminine mannerisms? (α=0.88) |
| **Implementation Outcomes** | |
| **Feasibility:** Recruitment yield | % of eligible HBC families who decide to participate |
| **Feasibility:** Peer Support Evaluation Inventory (PSEI)- Satisfaction Subscale (15-item) | I would recommend this type of support to another family  Receiving support from my family member was convenient for me. (0.96) |
| **Acceptability:** Completion rate | Proportion of participants who complete Our Family Our Voices |
| **Acceptability**: PSEI-supportive interactions^110^(15-items) | My family member accepted me for who I was (α=0.95) |
| **Acceptability:** PSEI-Relationship quality^110^ (30-items) | My family members were dependable (α=0.96) |

**3.7 STRENGTHS AND FUTURE DIRECTIONS.** The proposed study will test a novel adaptation of an intervention that integrates HIV risk reduction with family-based asset-building to increase HIV testing and engagement in medical care among HBC family members. The results of the formative research, including the pilot trial, will contribute to the evidence base regarding the development of HIV status-neutral interventions that respond to the diversity and complexities of families that are not themselves segregated by health status. The importance of asset-building to facilitate resilience as a part of the domestic HIV response is recognized as a critical missing component that is welcomed by key national stakeholders in the HBC (see letters of support). Based on findings from the pilot CRCT, we will finalize a protocol for a full CRCT application to conduct a definitive test of the efficacy of *OFOV* to improve HIV testing, PrEP use, care engagement and ARV treatment.

**BIBLIOGRAPHY**

1. Centers for Disease Control and Prevention. *Diagnoses of HIV infection in the United States and dependent areas, 2016: HIV Surveillance Report.* Atlanta: Author;2017.

2. Attia S, Egger M, Muller M, Zwahlen M, Low N. Sexual transmission of HIV according to viral load and antiretroviral therapy: systematic review and meta-analysis. *Aids.* 2009;23(11):1397-1404.

3. Sorensen SW, Sansom SL, Brooks JT, et al. A mathematical model of comprehensive test-and-treat services and HIV incidence among men who have sex with men in the United States. *PLoS One.* 2012;7(2):e29098.

4. Buchacz K, Armon C, Tedaldi E, et al. Disparities in HIV Viral Load Suppression by Race/ethnicity among Men who Have Sex with Men in the HIV Outpatient Study. *AIDS Res Hum Retroviruses.* 2018.

5. Crepaz N, Dong X, Wang X, Hernandez AL, Hall HI. Racial and Ethnic Disparities in Sustained Viral Suppression and Transmission Risk Potential Among Persons Receiving HIV Care - United States, 2014. *MMWR Morbidity and mortality weekly report.* 2018;67(4):113-118.

6. Arnold EA, Rebchook GM, Kegeles SM. 'Triply cursed': racism, homophobia and HIV-related stigma are barriers to regular HIV testing, treatment adherence and disclosure among young Black gay men. *Culture, health & sexuality.* 2014;16(6):710-722.

7. Bailey MM. *Butch Queen Up in Pumps: Gender, Perfornance and Ballroom Culture in Detroit.* Ann Arbor, MI: University of Michigan Press; 2013.

8. Herbst JH, Beeker C, Mathew A, et al. The effectiveness of individual-, group-, and community-level HIV behavioral risk-reduction interventions for adult men who have sex with men: a systematic review. *American journal of preventive medicine.* 2007;32(4 Suppl):S38-67.

9. Herbst JH, Painter TM, Tomlinson HL, Alvarez ME, Centers for Disease C, Prevention. Evidence-based HIV/STD prevention intervention for black men who have sex with men. *Morbidity and mortality weekly report Surveillance summaries.* 2014;63 Suppl 1:21-27.

10. Wilton L, Herbst JH, Coury-Doniger P, et al. Efficacy of an HIV/STI prevention intervention for black men who have sex with men: findings from the Many Men, Many Voices (3MV) project. *AIDS Behav.* 2009;13(3):532-544.

11. Donenberg GR, Paikoff R, Pequegnat W. Introduction to the special section on families, youth, and HIV: family-based intervention studies. *J Pediatr Psychol.* 2006;31(9):869-873.

12. Pequegnat W, Bray JH. HIV/STD prevention interventions for coulpes and families: A review and introduction to the special issue. *Couple and Family Psychology Research.* 2012;1(2):79-93.

13. Weiner BJ, Lewis MA, Clauser SB, Stitzenberg KB. In search of synergy: strategies for combining interventions at multiple levels. *J Natl Cancer Inst Monogr.* 2012;2012(44):34-41.

14. Nelson LE, Wilton L, Agyarko-Poku T, et al. Predictors of Condom Use among Peer Social Networks of Men Who Have Sex with Men in Ghana, West Africa. *PLoS One.* 2015;10(1):e0115504.

15. Maina G, Strudwick G, Lalani Y, Boakye F, Wilton L, Nelson LE. Characterizing the Structure and Functions of Social Networks of Men Who Have Sex with Men in Ghana, West Africa: Implications for Peer-Based HIV Prevention. *J Assoc Nurses AIDS Care.* 2017.

16. Arnold EA, Bailey MM. Constructing Home and Family: How the Ballroom Community Supports African American GLBTQ Youth in the Face of HIV/AIDS. *J Gay Lesbian Soc Serv.* 2009;21(2-3):171-188.

17. Wingood GM, DiClemente RJ. The ADAPT-ITT model: a novel method of adapting evidence-based HIV Interventions. *J Acquir Immune Defic Syndr.* 2008;47 Suppl 1:S40-46.

18. Nelson LE, Ogunbajo A, Abubakar MR, et al. Design of a culturally grounded HIV/STI prevention intervention for men who have sex with men in Ghana, West Africa: An application of the ADAPT-ITT model. *Manuscript in preparation.* 2019.

19. Birkhead GS, Riser MH, Mesler K, Tallon TC, Klein SJ. Youth development is a public health approach. Introduction. *J Public Health Manag Pract.* 2006;Suppl:S1-3.

20. Riser MH, Mesler K, Tallon TC, Birkhead GS. New York State's "Assets Coming Together (ACT) for Youth": a statewide approach effects community change. *Journal of public health management and practice : JPHMP.* 2006;Suppl:S41-47.

21. Scales PC. Reducing risks and building developmental assets: essential actions for promoting adolescent health. *J Sch Health.* 1999;69(3):113-119.

22. Prejean J, Song R, Hernandez A, et al. Estimated HIV incidence in the United States, 2006-2009. *PLoS One.* 2011;6(8):e17502.

23. Egan JE, Frye V, Kurtz SP, et al. Migration, neighborhoods, and networks: approaches to

understanding how urban environmental conditions affect syndemic adverse health outcomes among gay, bisexual and other men who have sex with men. *AIDS Behav.* 2011;15 Suppl 1:S35-50.

24. Fields EL, Bogart LM, Smith KC, Malebranche DJ, Ellen J, Schuster MA. HIV risk and perceptions of masculinity among young black men who have sex with men. *The Journal of adolescent health : official publication of the Society for Adolescent Medicine.* 2012;50(3):296-303.

25. Jeffries WLt, Marks G, Lauby J, Murrill CS, Millett GA. Homophobia is associated with sexual behavior that increases risk of acquiring and transmitting HIV infection among black men who have sex with men. *AIDS Behav.* 2013;17(4):1442-1453.

26. Kelly JA, St Lawrence JS, Amirkhanian YA, et al. Levels and predictors of HIV risk behavior among Black men who have sex with men. *AIDS education and prevention : official publication of the International Society for AIDS Education.* 2013;25(1):49-61.

27. Koblin BA, Mayer KH, Eshleman SH, et al. Correlates of HIV acquisition in a cohort of Black men who have sex with men in the United States: HIV prevention trials network (HPTN) 061. *PLoS One.* 2013;8(7):e70413.

28. Tieu HV, Spikes P, Patterson J, et al. Sociodemographic and risk behavior characteristics associated with unprotected sex with women among black men who have sex with men and women in New York City. *AIDS Care.* 2012;24(9):1111-1119.

29. Millett GA, Jeffries WLt, Peterson JL, et al. Common roots: a contextual review of HIV epidemics in black men who have sex with men across the African diaspora. *Lancet.* 2012;380(9839):411-423.

30. Millett GA, Peterson JL, Flores SA, et al. Comparisons of disparities and risks of HIV infection in black and other men who have sex with men in Canada, UK, and USA: a meta-analysis. *Lancet.* 2012;380(9839):341-348.

31. Levy ME, Wilton L, Phillips G, 2nd, et al. Understanding Structural Barriers to Accessing HIV Testing and Prevention Services Among Black Men Who Have Sex with Men (BMSM) in the United States. *AIDS Behav.* 2014.

32. Magnus M, Kuo I, Phillips G, 2nd, et al. Elevated HIV prevalence despite lower rates of sexual risk behaviors among black men in the District of Columbia who have sex with men. *AIDS Patient Care STDS.* 2010;24(10):615-622.

33. Sember R. Live to be legend. In: Mieseen M, Phillips A, eds. *Actors, Agents and Attendants Caring Culture: Art, Architecture and the Politics of Public Health.* Sternberg Press & SKOR Foundation for Art and Public Domain; 2011:191-202.

34. Gone JP. "We never was happy living like a Whiteman" : mental health disparities and the postcolonial predicament in American Indian communities. *American journal of community psychology.* 2007;40(3-4):290-300.

35. Ironson G, Stuetzle R, Ironson D, et al. View of God as benevolent and forgiving or punishing and judgmental predicts HIV disease progression. *J Behav Med.* 2011;34(6):414-425.

36. Moore M. Articulating a politics of multiple identities: LGBT sexuality and inclusion in Black community life. *DuBois Review.* 2010:1-20.

37. Nelson LE, Wilton L, Zhang N, et al. Childhood Exposure to Religions With High Prevalence of Members Who Discourage Homosexuality Is Associated With Adult HIV Risk Behaviors and HIV Infection in Black Men Who Have Sex With Men. *American journal of men's health.* 2017;11(5):1309-1321.

38. Nelson LE, Wilton L, Moineddin R, et al. Economic, Legal, and Social Hardships Associated with HIV Risk among Black Men who have Sex with Men in Six US Cities. *J Urban Health.* 2016;93(1):170-188.

39. Rivera Colon E. *Getting life in two worlds: Power and prevention in the New York City House Ball community*, Rutgers University; 2009.

40. Murrill CS, Liu KL, Guilin V, et al. HIV prevalence and associated risk behaviors in New York City's house ball community. *American journal of public health.* 2008;98(6):1074-1080.

41. Sanchez T, Finlayson T, Murrill C, Guilin V, Dean L. Risk behaviors and psychosocial stressors in the new york city house ball community: a comparison of men and transgender women who have sex with men. *AIDS Behav.* 2010;14(2):351-358.

42. Macapagal K, Bhatia R, Greene GJ. Differences in Healthcare Access, Use, and Experiences Within a Community Sample of Racially Diverse Lesbian, Gay, Bisexual, Transgender, and Questioning Emerging Adults. *LGBT Health.* 2016;3(6):434-442.

43. Whitfield DL, Walls NE, Langenderfer-Magruder L, Clark B. Queer is the new Black? Noe so much: Racial disparities in anti-:GBTQ discrimination. *Journal of Gay & Lesbian Social Services.*

2014;26(4):426-440.

44. Chmielewski JF, Belmonte KM, Fine M, Stoudt BG. Intersectional inquiries with LGBTQ and gender nonconforming yout of color: Participatort research on discipline disparities at the race/sexuality/gender nexus. In: Skiba R, Mediratta K, Rausch M, eds. *Inequality in School Discipline.* New York: Palgrave Macmillan; 2016.

45. Search Institute. *Developmental assets among LGBT youth.* Minneapolis, MN: Search Institute;2018.

46. Nelson LE, Walker JJ, DuBois SN, Giwa S. Your blues ain't like mine: considering integrative antiracism in HIV prevention research with black men who have sex with men in Canada and the United States. *Nursing inquiry.* 2014.

47. Jones KT, Gray P, Whiteside YO, et al. Evaluation of an HIV prevention intervention adapted for Black men who have sex with men. *American journal of public health.* 2008;98(6):1043-1050.

48. Duarte-Velez Y, Bernal G, Bonilla K. Culturally adapted cognitive-behavior therapy: integrating sexual, spiritual, and family identities in an evidence-based treatment of a depressed Latino adolescent. *J Clin Psychol.* 2010;66(8):895-906.

49. Somerville GG, Diaz S, Davis S, Coleman KD, Taveras S. Adapting the popular opinion leader intervention for Latino young migrant men who have sex with men. *Aids Educ Prev.* 2006;18(4):137-148.

50. Fishbein M, Higgins DL, Rietmeijer C, Wolitski RJ, G CACDPR. Community-level HIV intervention in 5 cities: Final outcome data from the CDC AIDS community demonstration projects. *Am J Public Health.* 1999;89(3):336-345.

51. Hosek SG, Lemos D, Hotton AL, et al. An HIV intervention tailored for black young men who have sex with men in the House Ball Community. *AIDS Care.* 2015;27(3):355-362.

52. Harper GW, Bruce D, Hosek SG, Fernandez MI, Rood BA, HIV AMTN. Resilience ProcessesDemonstrated by Young Gay and Bisexual Men Living with HIV: Implications for Intervention. *Aids Patient Care St.* 2014;28(12):666-676.

53. Harper GW, Fernandez IM, Bruce D, Hosek SG, Jacobs RJ, Network AMT. The Role of Multiple Identities in Adherence to Medical Appointments Among Gay/Bisexual Male Adolescents Living with HIV. *Aids and Behavior.* 2013;17(1):213-223.

54. Harper GW, Lemos D, Hosek SG, HIV AMTN. Stigma Reduction in Adolescents and Young Adults Newly Diagnosed with HIV: Findings from the Project ACCEPT Intervention. *Aids Patient Care St* 2014;28(10):543-554.

55. Lemos D, Hosek SG, Bell M. Reconciling Reality With Fantasy: Exploration of the Sociocultural Factors Influencing HIV Transmission Among Black Young Men Who Have Sex With Men (BYMSM) Within the House Ball Community: A Chicago Study. *Journal of Gay & Lesbian Social Services.* 2015;27(1):64-85.

56. Rao D, Kekwaletswe TC, Hosek S, Martinez J, Rodriguez F. Stigma and social barriers to medicationadherence with urban youth living with HIV. *Aids Care-Psychological and Socio-Medical Aspects ofAids/Hiv.* 2007;19(1):28-33.

57. Roy M, Levasseur M, Dore I, et al. Looking for capacities rather than vulnerabilities: The moderating effect of health assets on the associations between adverse social position and health. *Prev Med* 2018;110:93-99.

58. Ginwright S, James T. From assets to agents of change: social justice, organizing, and youth development. *New Dir Youth Dev.* 2002(96):27-46.

59. White Hughto JM, Hidalgo AP, Bazzi AR, Reisner SL, Mimiaga MJ. Indicators of HIV-risk resilience among men who have sex with men: a content analysis of online profiles. *Sex Health.* 2016.

60. Toska E, Gittings L, Hodes R, et al. Resourcing resilience: social protection for HIV prevention amongst children and adolescents in Eastern and Southern Africa. *Afr J AIDS Res.* 2016;15(2):123-140.

61. McNair OS, Gipson JA, Denson D, Thompson DV, Sutton MY, Hickson DA. The Associations of Resilience and HIV Risk Behaviors Among Black Gay, Bisexual, Other Men Who Have Sex with Men (MSM) in the Deep South: The MARI Study. *AIDS Behav.* 2018;22(5):1679-1687.

62. Chakrapani V, Kaur M, Newman PA, Mittal S, Kumar R. Syndemics and HIV-related sexual risk among men who have sex with men in India: influences of stigma and resilience. *Cult Health Sex.* 2018:1-16.

63. Hatzenbuehler ML, Nolen-Hoeksema S, Dovidio J. How does stigma "get under the skin"?: the mediating role of emotion regulation. *Psychological science.* 2009;20(10):1282-1289.

64. Hatzenbuehler ML. How does sexual minority stigma "get under the skin"? A psychological mediation framework. *Psychol Bull.* 2009;135(5):707-730.

65. Dean HD, Fenton KA. Addressing social determinants of health in the prevention and control of HIV/AIDS, viral hepatitis, sexually transmitted infections, and tuberculosis. *Public Health Rep.* 2010;125 Suppl 4:1-5.

66. Auerbach J. Transforming social structures and environments to help in HIV prevention. *Health affairs.* 2009;28(6):1655-1665.

67. Auerbach JD, Parkhurst JO, Caceres CF. Addressing social drivers of HIV/AIDS for the long-term response: conceptual and methodological considerations. *Glob Public Health.* 2011;6 Suppl 3:S293-309.

68. Sallis JF, Owen N, Fisher EB, Glanz K, Rimer BK. Ecological models of health behavior. In: *Health Behavior and Health Education: Theory, Research, and Practice.* 4th ed. San Francisco, CA: Jossey-Bass; 2008:465-486.

69. Stokols D. Establishing and maintaing healthy environments: Towards a social ecology of health promotion. *American Psychologist.* 1992;47(1):6-22.

70. Eaton LA, Driffin DD, Kegler C, et al. The role of stigma and medical mistrust in the routine health care engagement of black men who have sex with men. *Am J Public Health.* 2015;105(2):e75-82.

71. Earnshaw VA, Reed NM, Watson RJ, Maksut JL, Allen AM, Eaton LA. Intersectional internalized stigma among Black gay and bisexual men: A longitudinal analysis spanning HIV/sexually transmitted infection diagnosis. *J Health Psychol.* 2019:1359105318820101.

72. Bowleg L. "Once You've Blended the Cake, You Can't Take the Parts Back to the Main Ingredients": Black Gay and Bisexual Men's Descriptions and Experiences of Intersectionality. *Sex Roles.* 2013;68(11-12):754-767.

73. Bernat DH, Resnick MD. Healthy youth development: science and strategies. *J Public Health Manag Pract.* 2006;Suppl:S10-16.

74. Hamilton SF. Youth development and prevention. *J Public Health Manag Pract.* 2006;Suppl:S7-9.

75. Rao D, Elshafei A, Nguyen M, Hatezbuehler ML, Frey S, Go V. A systematic review of multi-level stigma interventions: State of the science and future directions. *BMC Medicine.* 2019.

76. Lyons CE, Ketende S, Diouf D, et al. Potential Impact of Integrated Stigma Mitigation Interventions in Improving HIV/AIDS Service Delivery and Uptake for Key Populations in Senegal. *J Acquir Immune Defic Syndr.* 2017;74 Suppl 1:S52-S59.

77. Stangl AL, Lloyd JK, Brady LM, Holland CE, Baral S. A systematic review of interventions to reduce HIV-related stigma and discrimination from 2002 to 2013: how far have we come? *J Int AIDS Soc.* 2013;16(3 Suppl 2):18734.

78. Loutfy M, Tharao W, Logie C, et al. Systematic review of stigma reducing interventions for African/Black diasporic women. *J Int AIDS Soc.* 2015;18:19835.

79. Simoni JM, Nelson KM, Franks JC, Yard SS, Lehavot K. Are peer interventions for HIV efficacious? A systematic review. *AIDS Behav.* 2011;15(8):1589-1595.

80. Simoni JM, Franks JC, Lehavot K, Yard SS. Peer interventions to promote health: conceptual considerations. *Am J Orthopsychiatry.* 2011;81(3):351-359.

81. Peterson JL, Rintamaki LS, Brashers DE, Goldsmith DJ, Neidig JL. The forms and functions of peer social support for people living with HIV. *J Assoc Nurses AIDS Care.* 2012;23(4):294-305.

82. Nelson LE, Aaful G, Adu-Sarkodie Y, et al. HIV Empathy, Education & Empowerment (HIVE3): Feasibility and acceptability of mobile-app based peer support for HIV positive MSM. National HIV/AIDS Research Conference; 2018; Accra, Ghana.

83. Maina G, Strudwick G, Lalani Y, Boakye F, Wilton L, Nelson LE. Characterizing the Structure and Functions of Social Networks of Men Who Have Sex with Men in Ghana, West Africa: Implications for Peer-Based HIV Prevention. *J Assoc Nurses AIDS Care.* 2018;29(1):70-82.

84. Wheeler DP, Fields SD, Beauchamp G, et al. Pre-exposure prophylaxis initiation and adherence among Black men who have sex with men (MSM) in three US cities: results from the HPTN 073 study. *J Int AIDS Soc.* 2019;22(2):e25223.

85. Wheeler DP, Lucas J, Wilton L, et al. Building effective multilevel HIV prevention partnerships with Black men who have sex with men: experience from HPTN 073, a pre-exposure prophylaxis study in three US cities. *J Int AIDS Soc.* 2018;21 Suppl 7:e25180.

86. Hirshfield S, Downing MJ, Jr., Chiasson MA, et al. Evaluation of Sex Positive! A

87. Bond KT, Yoon IS, Houang ST, Downing MJ, Grov C, Hirshfield S. Transactional Sex, Substance Use, and Sexual Risk: Comparing Pay Direction for an Internet-Based US Sample of Men Who Have Sex with Men. *Sex Res Soc Policy.* 2019;16(3):255-267.

88. Freeman AE, Sullivan P, Higa D, et al. Perceptions of Hiv Self-Testing among Men Who Have Sex with Men in the United States: A Qualitative Analysis. *Aids Educ Prev.* 2018;30(1):47-62.

89. Frye V, Wilton L, Hirshfield S, et al. Preferences for HIV test characteristics among young, Black Men Who Have Sex With Men (MSM) and transgender women: Implications for consistent HIV testing. *PLoS One.* 2018;13(2):e0192936.

90. Koblin B, Hirshfield S, Chiasson MA, et al. Intervention to Match Young Black Men and Transwomen Who Have Sex With Men or Transwomen to HIV Testing Options (All About Me): Protocol for a Randomized Controlled Trial. *Jmir Res Protoc.* 2017;6(12).

91. Mizuno Y, Borkowf C, Hirshfield S, Mustanski B, Sullivan PS, MacGowan RJ. Age and race/ethnicityspecific sex partner correlates of condomless sex in an online sample of Hispanic/Latino, Black/African American, and White men who have sex with men. *Arch Sex Behav.* 2019. In press.

92. Wilton L, Chiasson MA, Nandi V, et al. Characteristics and Correlates of Lifetime Suicidal Thoughts and Attempts Among Young Black Men Who Have Sex With Men (MSM) and Transgender Women. *J Black Psychol.* 2018;44(3):273-290.

93. Yoon IS, Downing MJ, Teran R, et al. Sexual risk taking and the HIV care continuum in an online sample of men who have sex with men. *Aids Care-Psychological and Socio-Medical Aspects of Aids/Hiv.* 2018;30(7):921-929.

94. Nelson LE, Morrison-Beedy D, Kearney MH, Dozier A. Always, never, or sometimes: examining variation in condom-use decision making among Black adolescent mothers. *Research in nursing & health.* 2011;34(4):270-281.

95. Morrison-Beedy D, Cote-Arsenault D, Feinstein NF. Maximizing results with focus groups: moderator and analysis issues. *Appl Nurs Res.* 2001;14(1):48-53.

96. Cote-Arsenault D, Morrison-Beedy D. Maintaining your focus in focus groups: avoiding common mistakes. *Res Nurs Health.* 2005;28(2):172-179.

97. Mayring P. Qualitative content analysis. *Forum for Qualitative Social Research.* 2000;1(2).

98. Chiasson MA, Shaw FS, Humberstone M, Hirshfield S, Hartel D. Increased HIV disclosure three months after an online video intervention for men who have sex with men (MSM). *Aids Care-Psychological and Socio-Medical Aspects of Aids/Hiv.* 2009;21(9):1081-1089.

99. Hirshfield S, Chiasson MA, Joseph H, et al. An Online Randomized Controlled Trial Evaluating HIV Prevention Digital Media Interventions for Men Who Have Sex with Men. *Plos One.* 2012;7(10).

100. Hirshfield S, Downing MJ, Parsons JT, et al. Developing a Video-Based eHealth Intervention for HIVPositive Gay, Bisexual, and Other Men Who Have Sex with Men: Study Protocol for a Randomized Controlled Trial. *Jmir Res Protoc.* 2016;5(2).

101. Nelson LE, Morrison-Beedy D. Research team training: moving beyond job descriptions. *Applied nursing research : ANR.* 2008;21(3):159-164.

102. Klein JD, Sabaratnam P, Auerbach MM, et al. Development and factor structure of a brief instrument to assess the impact of community programs on positive youth development: The Rochester Evaluation of Asset Development for Youth (READY) tool. *J Adolescent Health.* 2006;39(2):252-260.

103. Sabaratnam P, Klein JD. Measuring youth development outcomes for community program

evaluationand quality improvement: Findings from dissemination of the Rochester Evaluation of Asset Development for Youth (READY) tool. *J Public Health Man.* 2006:S88-S94.

104. Ivers NM, Halperin IJ, Barnsley J, et al. Allocation techniques for balance at baseline in cluster randomized trials: a methodological review. *Trials.* 2012;13.

105. Peterson NA, Speer PW, McMillan DW. Validation of a Brief Sense of Community Scale: Confirmation of the principal theory of sense of community. *J Community Psychol.* 2008;36(1):61-73.

106. Smith BW, Dalen J, Wiggins K, Tooley E, Christopher P, Bernard J. The brief resilience scale: assessing the ability to bounce back. *Int J Behav Med.* 2008;15(3):194-200.

107. Steward WT, Herek GM, Ramakrishna J, et al. HIV-related stigma: adapting a theoretical framework for use in India. *Social science & medicine.* 2008;67(8):1225-1235.

108. Diaz RM, Ayala G, Bein E, Henne J, Marin BV. The impact of homophobia, poverty, and racism on the mental health of gay and bisexual Latino men: findings from 3 US cities. *American journal of public health.* 2001;91(6):927-932.

109. Logie CH, Newman PA, Chakrapani V, Shunmugam M. Adapting the minority stress model: associations between gender non-conformity stigma, HIV-related stigma and depression among men who have sex with men in South India. *Social science & medicine.*2012;74(8):1261-1268.

110. Dennis CL. The effect of peer support on postpartum depression: a pilot randomized controlled trial. *Canadian journal of psychiatry Revue canadienne de psychiatrie.* 2003;48(2):115-124.

111. Lyles CM, Crepaz N, Herbst JH, Kay LS, Team HAPRS. Evidence-based HIV behavioral prevention from the perspective of the CDC's HIV/AIDS Prevention Research Synthesis Team. *AIDS Educ Prev.*

2006;18(4 Suppl A):21-31.

112. Zou GY. A modified Poisson regression approach to prospective studies with binary data. *American journal of epidemiology.* 2004;159(7):702-706.
